# Task-Specific Organic Salts and Ionic Liquids Binary Mixtures: A Combination to Obtain 5-Hydroxymethylfurfural From Carbohydrates

**DOI:** 10.3389/fchem.2019.00134

**Published:** 2019-03-21

**Authors:** Salvatore Marullo, Carla Rizzo, Francesca D'Anna

**Affiliations:** Dipartimento di Scienze e Tecnologie Biologiche, Chimiche e Farmaceutiche, Università degli Studi di Palermo, Palermo, Italy

**Keywords:** ionic liquids, carbohydrate conversion, 5-hydroxymethylfurfural, sustainable chemistry, task-specific organic salts

## Abstract

The increase in energy demand and depletion of fossil fuels are among major issues of modern society. Valorization and transformation of raw materials in products of industrial value represent a challenge. This justifies the growing interest of scientific research toward the identification of suitable media and methodologies able to pursue above goals, paying attention to matter of sustainability. On this subject, we studied sulfonic-acid functionalized diimidazolium salts as catalysts for the conversion of fructose and sucrose to 5-hydroxymethylfurfural (5-HMF) in an ionic liquid mixture. In general, using these salts allowed us to obtain 5-HMF in good yields from both substrates in mild conditions. Indeed, at 60°C and in the presence of 20 mol% of catalyst, 5-HMF yields of 60 and 30% were obtained from fructose and sucrose, respectively. The catalytic system was recycled and used up to six times observing no appreciable loss in yield for the first four cycles. Moreover, we gathered mechanistic information by *in situ* ^1^H NMR monitoring the dehydration of fructose. To dissect the role of acidity on the reaction, we determined the Hammett acidity function of each salt. Comparison of these results with yields and reactivity observed in the presence of related monocationic salts and with a dicationic salt bearing only one sulfonic acid group, allowed stating that the reactivity observed is the result of the combined action of acidity and structural features of the catalysts. Overall, the approach proposed here could contribute to pave the way to increase sustainability in the raw material valorization processes.

## Introduction

The steady increase in energy demand in modern society has led researchers to tackle pressing challenges like global warming and the depletion of petroleum feedstocks together with the urgent need to reduce the generation of waste from industrial processes. As a result, the replacement of fossil fuels and energy sources with renewable alternatives features prominently in present-day chemical research. In this respect, the chemical conversion of vegetable biomasses in value-added chemicals represents a viable route to pursue these goals (Zhou et al., 2011; Brun et al., 2017; Den et al., 2018). Indeed, vegetable biomass is a naturally abundant, readily available resource posing no environmental risk. Deriving from agricultural waste, vegetable biomass also provides a renewable resource at a negligible cost (Pierre, 2008). Accordingly, efficient conversion of biomass gives access to a wide range of chemical platforms, i.e., key intermediates for the synthesis of industrially relevant products. These include ethanol, succinic and 3-hydroxypropionic acids, levulinic acid, isoprene and so on (Chinnappan et al., 2016; Mika et al., 2018). In this context, 5-hydroxymethylfurfural (5-HMF) and its derivatives are among the most important chemical platforms obtained from the conversion of the carbohydrate rich fraction of biomasses (Rout et al., 2016). 5-HMF is a key intermediate to the production of solvents, biofuels and polymers (Zhang and Dumont, 2017) and is in principle easily obtained from the acid-catalyzed dehydration of fructose. However, obtaining 5-HMF from biomass is a more challenging task because the carbohydrate-based fraction of biomass is overwhelmingly richer in glucose-based materials. Hence, an intermediate step of isomerization of the glucose obtained by the breakdown of the biomass into fructose is required. The conventional methods for the production of 5-HMF require harsh reaction conditions with high temperatures and strong mineral acids like H_2_SO_4_ or metal based Lewis acids like CrCl_3_ (Chheda et al., 2007).

Frequently, the use of harsh reaction conditions combined with corrosive or metal based catalysts is associated to the occurrence of undesired side reactions, entailing further problems when the greenness of the process is evaluated. The growing concerns for sustainability of chemical processes, embodied by the principles of Green Chemistry (Anastas and Eghbali, 2010) have led researchers to explore milder reaction conditions, shifting to safer and less environmentally impacting heterogeneous catalysts (De et al., 2016) such as zeolites (Taarning et al., 2011; Perego et al., 2017) and ionic exchange resins (Ramírez et al., 2017). Furthermore, applying analogous criteria to the selection of solvents has evidenced that non-conventional media such deep eutectic solvents (Vigier et al., 2015; Zuo et al., 2017) and ionic liquids (ILs) (Tim et al., 2011; Yoo et al., 2017; Zhang et al., 2017; Mika et al., 2018) can be suitable to carry out this process. ILs are organic salts with a low melting point (< 100°C) and often liquid at room temperature (Plechkova and Seddon, 2008; Hallett and Welton, 2011). They provide unique fully ionic reaction media with a distinct nanostructure (Dupont, 2011; Hayes et al., 2015). Regarding the topic of biomass conversion, some ILs stand out for their remarkable ability of solubilizing cellulose, the fraction of biomass richest in carbohydrates, by disrupting intra- and intermolecular hydrogen bonds (Sun et al., 2011).

Along these premises, in the framework of our interest in studying ILs properties and applications, (D'Anna et al., 2009b,c, 2011, 2013). We investigated the conversion of carbohydrates into 5-HMF, using IL binary mixtures as solvent system and the acidic ion exchange resin Amberlyst 15 as catalyst (D'Anna et al., 2014). We tested different IL binary mixtures, finding the 1-butyl-3-methylimidazolium chloride/tetrafluoroborate ([bmim][Cl]/[bmim][BF_4_]) mixture as the best solvent system for both mono (fructose and glucose) and disaccharides (sucrose) dehydration, under silent conditions as well as ultrasound irradiation. In particular, we demonstrated that yield in 5-HMF changed as a function of binary mixture composition, with the best results obtained at X_Cl_ ≈ 0.5. In the light of the above results, with the aim to use more active catalysts under milder conditions, we herein report on fructose and sucrose dehydration in the same IL mixture, testing the catalytic efficiency of acid-functionalized diimidazolium salts.

Endowing imidazolium or other ILs with catalytic moieties gives rise to the so-called Task Specific Ionic Liquids (TSILs) (Giernoth, 2010; Yue et al., 2011). A wide range of functionalities can be easily incorporated in ILs, (Chiappe and Pomelli, 2014) and in this context, we have successfully applied this approach for the obtainment of TSILs able to promote different reactions from the synthesis of aryl azides (D'Anna et al., 2008) to Michael addition (Rizzo et al., 2016a) and heterocyclic rearrangements (Rizzo et al., 2014). In particular, using acid-functionalized TSILs for the conversion of biomass can be a viable route to obtain value added chemicals under mild homogeneous conditions.

Functionalization of ILs with sulfonic acid groups is a widely used approach to enhance their acidity (Amarasekara, 2016; Chiappe et al., 2017) and promote the conversion of carbohydrates avoiding metal-based catalysts and mineral acids. However, to date the vast majority of the reports feature monocationic ILs, while the use of dicationic ILs has been less explored (Jadhav et al., 2012, 2014; Liu et al., 2015; Yaman et al., 2017). To the best of our knowledge only one report deals with supported sulfonic acid functionalized dicationic ILs as catalyst for the conversion of carbohydrates (Liu et al., 2015).

Dicationic ILs make for environmentally benign catalysts with high thermal stability (Fang et al., 2011). Moreover, dicationic ILs are generally less miscible with conventional organic solvents than corresponding monocationic ones (D'Anna and Noto, 2014). This could enable easier recycling of the solvent/catalyst system improving the whole eco-compatibility of studied processes.

**Figure 1:**
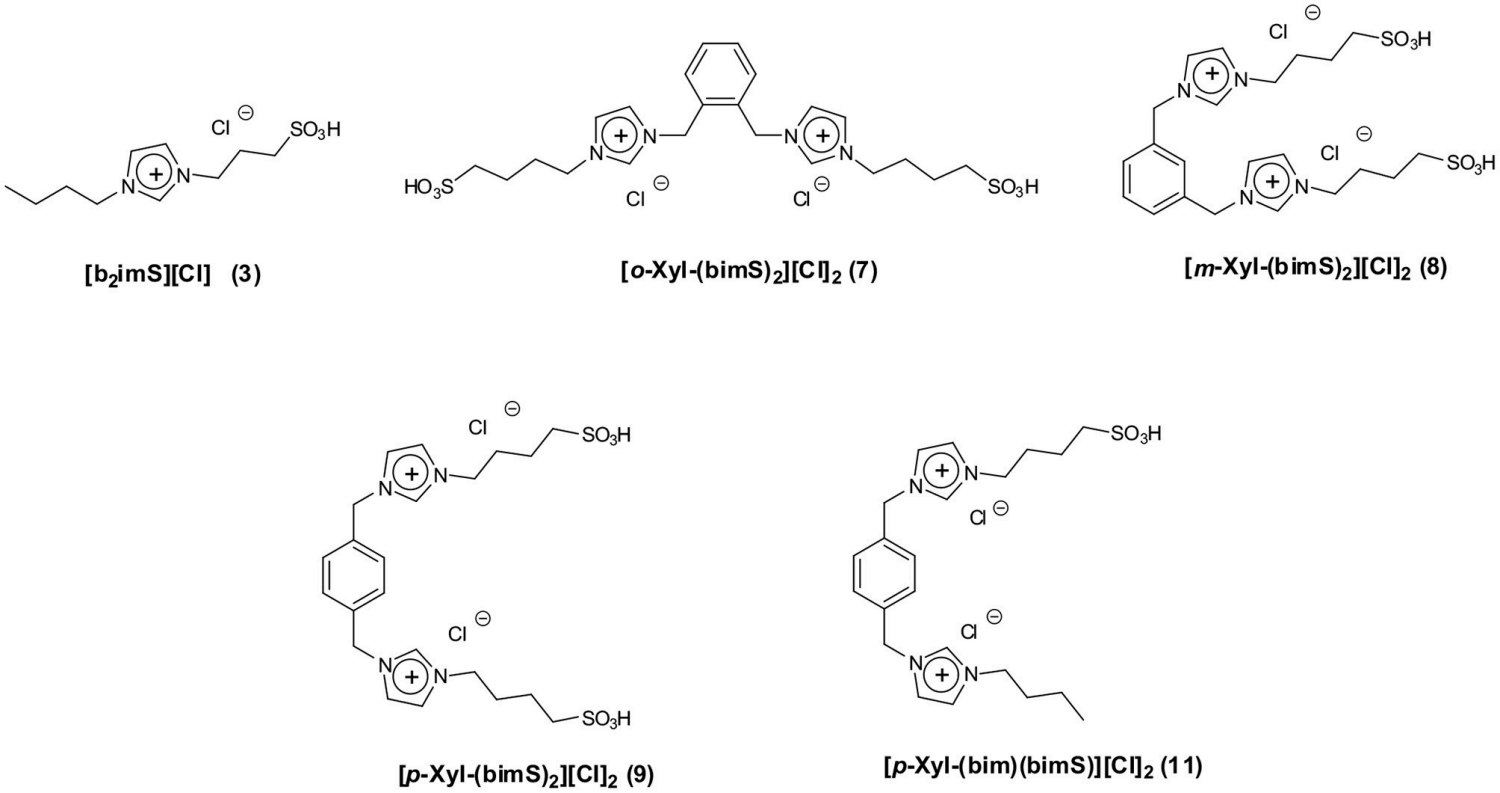
Structures of sulfonic acid-functionalized mono- and dicationic imidazolium salts.

Bearing this in mind, we herein report the use of some sulfonic acid-functionalized diimidazolium salts bearing 1,2- 1,3- and 1,4-xylilene spacers interposed between the charged heads (Figure 1) as catalysts for the conversion of fructose and sucrose into 5-HMF.

We tested all the isomers to evaluate how the different distance between charged heads, that could affect cation conformation, (D'Anna et al., 2009a, 2013) acts on the catalytic efficiency of our salts. Data collected in the presence of diimidazolium salts were compared with the ones obtained using 1-butyl-3-sulfobutyl-imidazolium chloride (**[b**_**2**_**imS][Cl]**, Figure 1) to verify whether the catalytic activity of dicationic salts derives simply from the combination of two acidic sites.

**Figure 2:**
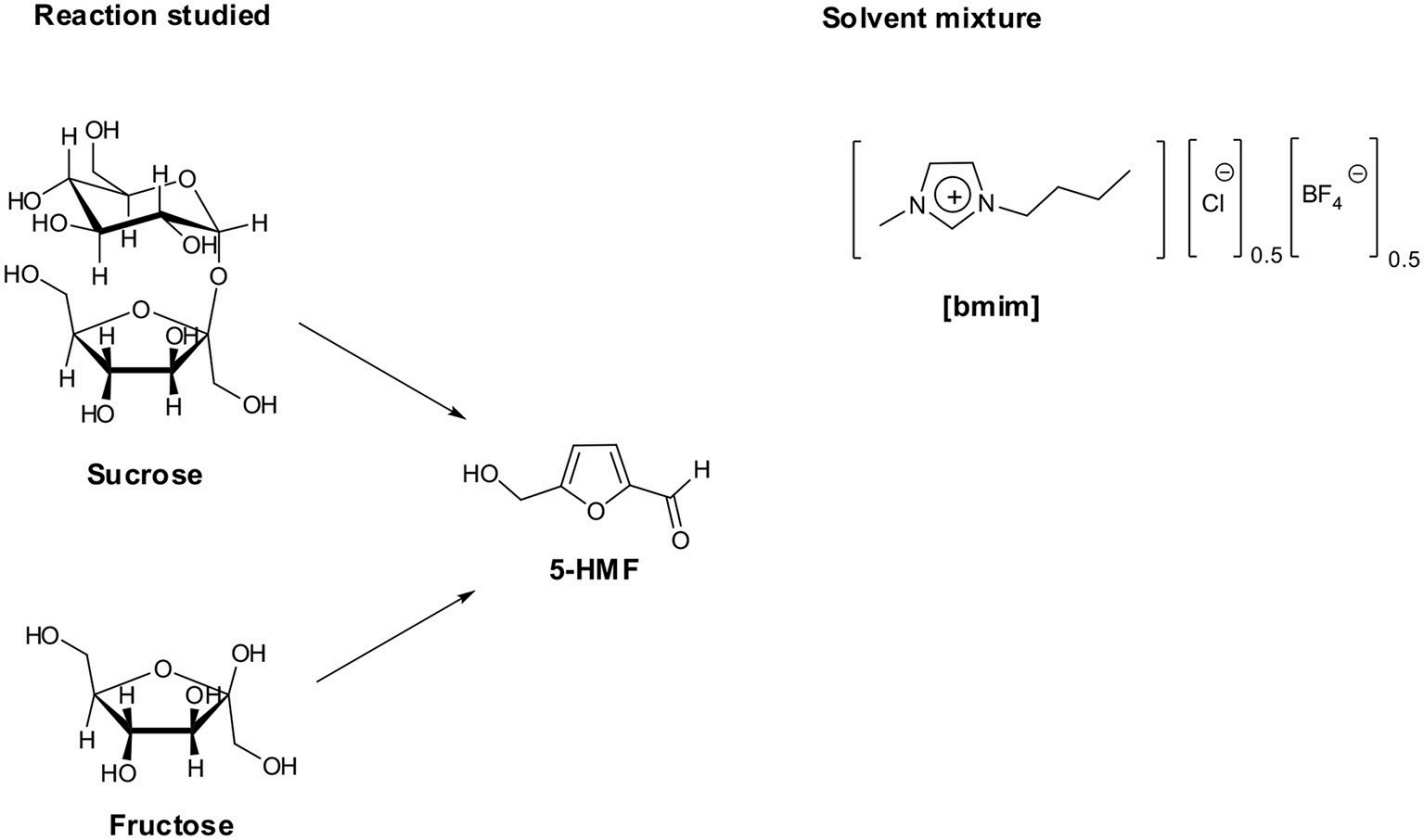
Reaction studied and IL mixture used as solvent.

Moreover, to gain information on the effect exerted by the presence of a charged head devoid of sulfonic acid functionality, we also tested the salt 1-[1′-methylene-3′-butylimidazolium]-4-[1′-methylene-3′-sulfobutylimidazolium]benzene dichloride (**[*p*-Xyl-(bim)(bimS)][Cl]**_**2**_, Figure 1). All reactions were carried out at 60°C (Figure 2).

To find the best reaction conditions, we analyzed the effect of operating parameters such as the amount of catalyst and reaction time. Finally, we determined the acid strength of the catalyst by means of the Hammett acidity function *H*_0_ (Hammett and Deyrup, 1932) to dissect its role on the reaction outcome.

The results collected in this paper highlight the suitability of ILs binary mixtures to promote carbohydrate conversion into 5-HMF. Furthermore, they shed light on the good performance of acidic sulfonate TSILs whose action results from the balance of different factors besides acidity. The IL-based catalytic system could be recycled up to six times and mechanistic information were gathered by *in situ* ^1^H-NMR monitoring the reaction.

## Materials and Methods

### Materials

Commercially available 1-butylimidazole, 1-chlorobutane, 1,4-butanesultone, acetonitrile, fructose, and sucrose were used without further purification. ILs, 1-butyl-3-methylimidazolium tetrafluoroborate [bmim][BF_4_] and 1-butyl-3-methylimidazolium chloride [bmim][Cl] obtained from commercial sources, were dried at 70°C in a vacuum line for 2 h. Then, they were lyophilized for 48 h at 0.06 mbar, and stored in a desiccator under argon and over calcium chloride prior each use. 1,2-, 1,3- and 1,4-bis(imidazolemethyl)benzene were prepared following reported procedures (D'Anna et al., 2012; Rizzo et al., 2016b).

The NMR spectra were recorded by using Bruker 300 or 250 MHz. ESI-MS spectra were recorded on a Q-TOF spectrometer in the positive ionization mode. Samples were prepared by dissolving suitable amounts of salts in methanol.

### 1,2-bis(Imidazolemethyl)Benzene (4)

1,2-bis(imidazolemethyl)benzene **(4)** was prepared according to a published procedure (D'Anna et al., 2013).

Imidazole (3.0 g, 0.045 mol) and potassium hydroxide (4.9 g, 0.09 mol) were dissolved in acetonitrile (300 mL) and stirred for 2 h at room temperature. Then, α,α'-*o*-dibromoxylene (5.9 g, 0.022 mol) was added to the mixture. The reaction mixture was stirred at room temperature for 1.5 h. The mixture was filtered through a Hirsch funnel to remove insoluble salts and the filtrate was concentrated in vacuo at 40°C. The residue was dissolved in CHCl_3_ (1 L) and washed with water (4 × 150 mL) until the aqueous layer was neutral to p*H* paper. The organic layer was dried over anhydrous Na_2_SO_4_ and removal of the solvent afforded a pale yellow solid (2.4 g, 44%).

### Synthesis of the Sulfonic Acid Functionalized Imidazolium Salts

#### 1-Butyl-3-Sulfobutyl Imidazolium Chloride [b_2_imS][Cl] (3)

To a stirred solution of 1-butylimidazole **(1)** (1g) in acetonitrile, 1.65 mL of 1,4-butanesultone **(2)** were added dropwise and the resulting mixture was stirred at 100°C for 24 h. Removal of the solvent afforded a yellow solid which was washed with several portions of acetone. The resulting solid was filtered off and dissolved in 20 mL of methanol. Then, the stoichiometric amount of HCl (37% w:w in water) was added and the resulting mixture was stirred at room temperature for 1 h. Subsequent removal of the solvent afforded a colorless oil (1.5 g, yield 68%).

### General Procedure for the Synthesis of Symmetrically Substituted Diimidazolium Salts

To a stirred solution of the suitable bis(imidazolemethyl)benzene (1g, 4.2 mmol) in 30 mL acetonitrile, 1,4-butanesultone **(2)** was added (1.71 g, 12.6 mmol) and the resulting mixture was refluxed under inert atmosphere for 72 h. The reaction mixture was cooled down and acetone was added until complete precipitation of a colorless solid. The resulting solid was filtered, washed with acetone and then dissolved in methanol. To the resulting solution, the stoichiometric amount of HCl (37% w:w in water) was added and the mixture obtained was stirred at room temperature for 1 h. Subsequent removal of the solvent afforded the diimidazolium salts as colorless solids (All ^1^H and ^13^C NMR and mass spectra are reported in **Figures S2, S4**).

#### 1,2-Bis-[1′-Methylene-3′-Sulfobutylimidazolium]Benzene Dichloride [o-Xyl-(bimS)_2_][Cl]_2_ (7)

Yield: 42%. Colorless solid; m. p.: 106°C. ^1^H NMR (250 MHz, D_2_O); δ (ppm): 8.64 (s, 2H, exch); 7.46 (m, 4H); 7.29 (m, 4H); 5.38 (s, 4H); 4.08 (t, *J* = 7.5 Hz, 4H); 2.78 (t, *J* = 7.5 Hz, 4H); 1.83 (quin, *J* = 7.5 Hz, 4H); 1.58 (quin, *J* = 7.5 Hz, 4H). ^13^C NMR (250 MHz, D_2_O); δ (ppm): 134.2; 133.6; 133.3; 125.5; 125.2; 52.6 (2C, overlapped); 51.9; 30.6; 23.6. *ESI (m/z)*: 511 [C-H]^+^; 533 [C-2H+Na]^+^.

#### 1,3-Bis-[1′-Methylene-3′-Sulfobutylimidazolium]Benzene Dichloride [m-Xyl-(bimS)_2_][Cl]_2_ (8)

Yield: 100%. Colorless solid; m. p.: 74°C. ^1^H NMR (250 MHz, D_2_O); δ (ppm): 8.78 (s, 2H, exch); 7.39 (m, 8H); 5.32 (s, 4H); 4.16 (t, *J* = 7.5 Hz, 4H); 2.82 (t, *J* = 7.5 Hz, 4H); 1.95 (quin, *J* = 7.5 Hz, 4H); 1.61 (quin, *J* = 7.5 Hz, 4H). ^13^C NMR (250 MHz, D_2_O); δ (ppm): 138.3; 137.5; 133.0; 131.9; 130.8; 125.4; 55.1; 52.7; 51.8; 30.6; 23.6. *ESI (m/z)*: 511 [C-H]^+^; 533 [C-2H+Na]^+^.

#### 3,3′-Di-Sulfobutyl-1,1′-(1,4-Phenylenedimethylene)Diimidazolium Dichloride [p-Xyl-(bimS)_2_][Cl]_2_ (9)

Yield: 100%. Colorless solid; m. p.: 112°C. ^1^ NMR (250 MHz, CD_3_OD); δ (ppm): 9.14 (s, 2H); 7.67 (d, *J* = 9.0 Hz, 4H); 7.50 (s, 4H); 5.45 (s, 4H); 4.28 (t, *J* = 7.5 Hz, 4H); 2.82 (t, *J* = 7.5 Hz, 4H); 2.02 (quin, *J* = 7.5 Hz, 4H); 1.75 (quin, *J* = 7.5 Hz, 4H). ^13^C NMR (250 MHz, CD_3_OD); δ (ppm): 135.5 (2C, overlapped), 134.6, 129.3, 122.6, 105.4, 52.3, 49.9, 49.6, 28.1, 20.8. *ESI (m/z)*: 511 [C-H]^+^; 533 [C-2H+Na]^+^.

#### 1-[1′-Methyleneimidazole]-4-[1′-Methylene-3′-Sulfobutylimidazolium]Benzene Inner Salt (10)

To a stirred solution of 1,4-bis(imidazolemethyl)-benzene **(6)** (1 g, 4.2 mmol) in acetonitrile (60 mL), 1,4-butanesultone **(2)** (0.57 g, 4.2 mmol) was added dropwise. The reacting mixture was kept at 90°C, for 72 h, under stirring. Subsequently, the solvent was removed and the residue was purified by flash chromatography on aluminum oxide eluting with ethyl acetate/methanol/water 2/2/1 mixture, affording 0.72 g of a yellow solid. Then, it was dissolved in 5 mL of methanol and, upon adding acetone, a white precipitate was obtained. The resulting solid was filtered off and further washed with acetone, then dissolved in anhydrous dichloromethane and treated with activated charcoal at room temperature overnight. The mixture was finally filtered through a pad of neutral aluminum oxide and solvent removal afforded 0.69 g of a colorless solid (^1^H and ^13^C NMR spectra are reported in Figure S2).

Yield: 44%. Colorless solid; m. p.: 105°C; ^1^H NMR (250 MHz, CD_3_OD); δ (ppm): 7.75 (s, 1H); 7.66 (d, *J* = 2.5 Hz, 1H); 7.61 (d, *J* = 2.5 Hz, 1H); 7.42 (d, *J* = 8.0 Hz, 2H); 7.32 (d, *J* = 8.0 Hz, 2H); 7.11 (s, 1H); 6.97 (s, 1H); 5.41 (s, 2H); 5.22 (s, 2H); 4.26 (t, *J* = 7.5 Hz, 2H); 2.83 (t, *J* = 7.5 Hz, 2H); 2.01 (quin, *J* = 7.5 Hz, 2H); 1.76 (quin, *J* = 7.5 Hz, 2H). ^13^C NMR (250 MHz, CD_3_OD); δ (ppm): 142.9; 138.4; 132.8; 132.6; 127.4; 127.1; 124.1; 62.2; 56.9; 54.4; 32.9; 25.9.

#### 1-[1′-Methylene-3′-Butylimidazolium]-4-[1′-Methylene-3′-Sulfobutylimidazolium]Benzene Dichloride [p-Xyl-(bim)(bimS)][Cl]_2_ (11)

1-chlorobutane (0.5 g, 5.4 mmol) and (**10**) (0.67 g, 1.8 mmol) were dissolved in 20 mL of 2-propanol and the resulting mixture was heated, at 90°C, for 72 h. Subsequently, the solvent was removed and the residue was purified by flash chromatography on aluminum oxide, eluting with ethyl acetate/methanol/water 2/2/1 mixture affording 0.54 g of a pale yellow solid. This latter was dissolved in 5 mL methanol and added with 90 μL of hydrochloric acid (37% w:w in water). Finally, removal of the solvent afforded 0.58 g of a pale yellow solid (^1^H and ^13^C NMR, and mass spectra are reported in Figures S2, S4).

Yield: 64%. Pale yellow solid; m. p.: 109°C. ^1^H NMR (250 MHz, CD_3_OD); δ (ppm): 9.16 (s, 1H, exch); 9.08 (s, 1H, exch); 7.78 (m, 4H); 7.51 (m, 4H); 5.49 (m, 8H); 4.28 (m, 6H); 3.31 (m, 2H); 2.82 (t, *J* = 7.5 Hz, 4H); 2.04 (quin, *J* = 7.5 Hz, 4H); 1.89 (quin, *J* = 7.5 Hz, 2H); 1.73 (quin, *J* = 7.5 Hz, 4H); 1.38 (sext, *J* = 7.5 Hz, 2H); 0.98 (t, *J* = 7.5 Hz, 3H). ^13^C NMR (250 MHz, CD_3_OD); δ (ppm): 150.5; 149.4 (2C); 149.3; 143.5; 143.4; 137.1; 136.9; 136.8; 136.3; 134.4; 66.5; 64.3; 63.7; 63.5; 45.9; 42.6; 35.5; 33.3; 26.6. *ESI (m/z*): 431[C-H]^+^

### General Procedure for Conversion of Carbohydrate in 5-HMF

A suitable amount of carbohydrate (0.025 g) was weighed in a round-bottom flask containing 0.5 g of IL binary mixture [bmim][Cl]_0.5_[BF_4_]_0.5_. To favor carbohydrate dissolution, the mixture was stirred at 80°C for 30 min under argon. In all cases, the mixtures appeared homogeneous after this treatment.

For reactions carried out in conventional solvents, the substrates were dissolved in the suitable amount of solvent (500 μL) at room temperature.

After equilibration at the reaction temperature, the suitable amount of catalyst was added under argon atmosphere. The mixture was heated, at 60°C, under stirring. To monitor the amount of 5-HMF formed, a small aliquot of reaction mixture was withdrawn and diluted with methanol to reach a 5-HMF concentration ranging from 7 · 10^−6^ M up to 7 · 10^−5^ M. Hydrolysis of the 5-HMF dimer was carried out by adding 250 μL of ultrapure water and stirring for 20 min at room temperature. The concentration of 5-HMF was determined from UV absorbance recorded at 277 nm on the basis of a calibration curve previously determined. The presence of 5-HMF in the reaction mixture, as single UV absorbing product, was further verified by means of TLC on silica gel by comparison with a standard sample (eluent: ethyl acetate/methanol 5:1, v/v). To determine the amount of unreacted carbohydrates, an aliquot of the reaction mixture was dissolved in methanol/water 80/20 v:v and injected in a HPLC system equipped with a SUPELCOSIL-C_18_ column, using methanol/water 80/20 as eluting mixture. However, in all chromatograms the peaks of carbohydrates were not distinguishable from those of the ILs component and their amounts could not be determined. Conversely, 5-HMF was eluted after a retention time t_R_ = 5.0 min. Yields in 5-HMF determined by HPLC were in agreement with those measured by UV-vis spectroscopy within ± 3%.

### Recycle of the IL

The reaction mixture was extracted under vigorous stirring with diethyl ether (4 × 20 mL), then the residual extraction solvent was removed by evaporation under reduced pressure at 70°C for 3 h. The resulting IL was then charged with fresh fructose and the reaction was carried out as described above. The amount of 5-HMF extracted was determined spectrophotometrically as described above.

### *In situ* ^1^H NMR Analysis

^1^H NMR spectra were recorded on a 300 MHz spectrometer at 60°C. Samples were prepared by mixing in a NMR tube, a solution containing 25 mg of fructose with a solution containing the suitable amount of **[*o*-Xyl-(bimS)**_**2**_**][Cl]**_**2**_, both in the IL binary mixture [bmim][Cl]_0.5_[BF_4_]_0.5_. The sample was rapidly transferred into the chamber of the spectrometer and spectra recorded at selected times. A coaxial capillary tube loaded with d_6_-DMSO was used for the external lock of the NMR magnetic field/frequency, and its signal at δ = 2.56 ppm was used as the ^1^H NMR external reference.

### Determination of the Acid Strength of the Salts (Hammett and Deyrup, 1932; Thomazeau et al., 2003)

The suitable amount of acidic imidazolium salt was added to a methanol solution of methyl orange (2 · 10^−4^ M). Upon adding the salts a color change in the solution was observed. The resulting solution was analyzed by UV-vis spectroscopy. The concentration of salts in the sample was 2 · 10^−3^ M for the salts (**3**) and (**11**) and 1 · 10^−3^ M for all the other salts. Acid strength was expressed by the *H*_0_ acidity function defined by Equation (1):

(1) $$\begin{array}{r} H_{0}=pK_{I}+log\left(\left[I\right]/\left[HI^{+}\right]\right) \end{array}$$

where p*K*_I_ is the logarithm of the dissociation constant of the indicator used, [I] and [HI^+^] are the concentration of the indicator and its conjugated acid, respectively. The [I]/[HI^+^] ratio was determined spectrophotometrically after the evaluation of the ε value of the indicator.

## Results and Discussion

### Synthesis of the Acidic Imidazolium Salts

**Figure 3:**
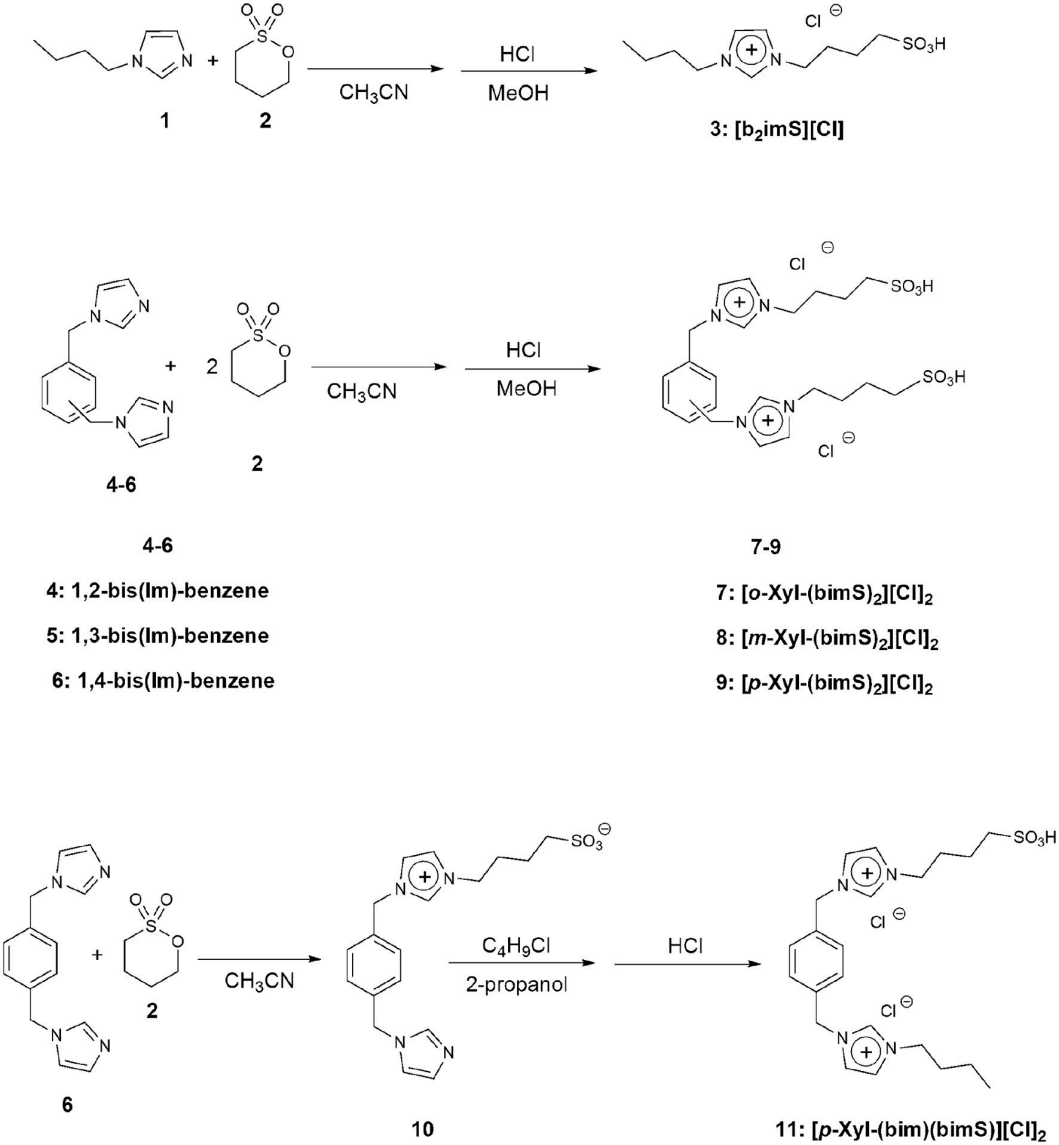
Synthetic steps for the sulfonic acid functionalized imidazolium salts employed.

All the salts considered in this work were prepared by modifications of a previously reported procedure (Ullah et al., 2016). The synthetic procedures followed are reported in Figure 3. In particular, the monocationic salt **[b**_**2**_**imS][Cl]** (**3**) was prepared by reacting 1-butylimidazole (**1**) with a stoichiometric amount of 1,4-butanesultone (**2**) followed by protonation with HCl. The symmetrically substituted salts **[*o*-Xyl-(bimS)**_**2**_**][Cl]**_**2**_, **[*m-*Xyl-(bimS)**_**2**_**][Cl]**_**2**_ and **[*p*-Xyl-(bimS)**_**2**_**][Cl]**_**2**_ (**7-9**) were prepared by following a two-step synthetic scheme. In the first step, the neutral diimidazole precursor (**4-6**) was reacted with excess of 1,4-butanesultone (**2**) to obtain the relevant sulfonate appended diimidazolium zwitterion, which in turn was treated with the stoichiometric amount of hydrochloric acid to yield the relevant sulfonic acid functionalized diimidazolium salt. The non-symmetrically substituted salt **[*p*-Xyl-(bim)(bimS)][Cl]**_**2**_ (**11**) was prepared through a three-step synthesis. In the first step 1,4-bis-(imidazolemethyl)-benzene (**6**) was reacted with a stoichiometric amount of 1,4-butanesultone (**2**) to afford the relevant sulfonate imidazolium zwitterion (**10**).

In a subsequent step, **10** was further alkylated with chlorobutane. Finally, protonation with hydrochloric acid afforded the diimidazolium salt bearing one sulfonic acid functionality. Full synthetic details are reported in the experimental section.

### Conversion of Carbohydrates in 5-HMF: Optimization of Reaction Conditions

Firstly, we searched for the possible catalytic activity of the IL binary mixture, performing both in the case of fructose and sucrose, the conversion in the absence of catalyst at 60°C. In both cases, we did not detect the formation of 5-HMF. Similar results were obtained using ethanol and DMSO as solvents. They were used to have a comparison with conventional organic solvents and chosen on the grounds of the eco-compatibility of the first one and the wide use of the latter in such kind of reactions.

**Figure 4:**
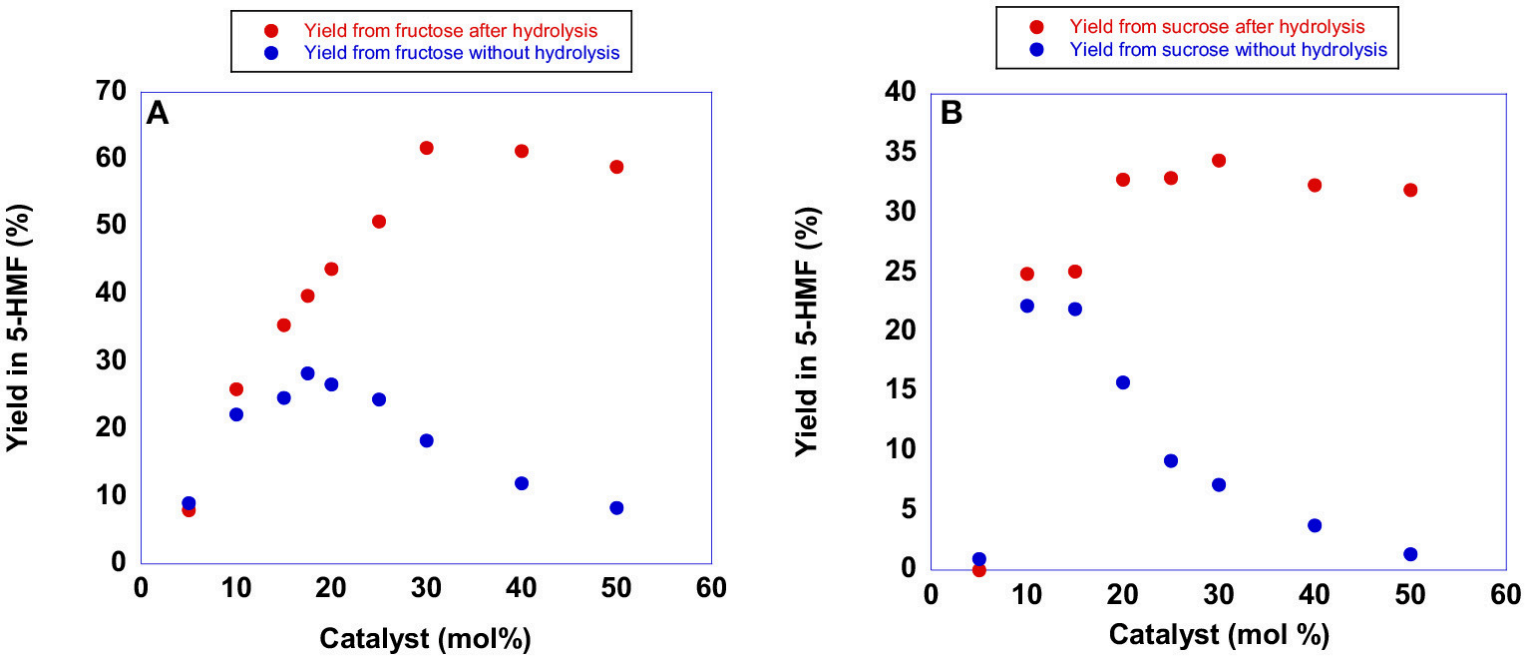
Plots of yields in 5-HMF from **(A)** fructose obtained at 60°C after 105 min and **(B)** sucrose obtained at 60°C after 150 min in the presence of increasing amounts of **[b**_**2**_**imS][Cl]**.

Subsequently, we set out the optimal amount of catalyst by performing the conversion of sucrose and fructose at 60 °C in the presence of increasing amounts of **[b**_**2**_**imS][Cl]**, ranging from 5 to 50 mol% with respect to the substrate. Yields in 5-HMF were determined spectrophotometrically (see experimental section for details). Plots of yields in 5-HMF as a function of the amount of **[b**_**2**_**imS][Cl]** are reported in Figure 4, while yield values are reported in Table S1.

In both cases, the suitable reaction time was chosen on the grounds of a kinetic investigation performed using 20 mol % of catalysts (see later).

Examination of the plots reported in Figure 4 (blue trace) revealed that the yield in 5-HMF increased with the amount of catalyst until reaching a maximum value at 20 mol % of catalyst. Further increasing the amount of **[b**_**2**_**imS][Cl]** led to a steady reduction in yields. Monitoring the reactions by TLC, we observed that the formation of 5-HMF was accompanied by the formation of a less polar by-product. Moreover, the formation of the by-product increased using higher amounts of **[b**_**2**_**imS][Cl]**.

We explained this finding by considering that under acidic conditions, 5-HMF can undergo dimerization forming 5,5′(oxy-bis(methylene))bis-2-furfural (Che et al., 2012; Galkin et al., 2016). Accordingly, the dimer can be converted back to 5-HMF simply by hydrolysis (Lewkowski, 2001). For this reason, we determined the yield in 5-HMF in both cases also after performing hydrolysis by adding 250 μL of water and stirring for 20 min prior to the spectroscopic determination. As can be seen from the plots, (red trace) hydrolysis of the dimer afforded back 5-HMF. Moreover, an amount of catalyst of 20 mol % was sufficient to obtain good yields in 5-HMF in both cases. Similar conclusions can be drawn by examining the results of analogous experiments in the presence of the other catalysts. The relevant plots are reported in Figure S1 and Tables S2, S3.

It is important to point out that the *p*-substituted salts, **[*p*-Xyl-(bimS)**_**2**_**][Cl]**_**2**_ and **[*p*-Xyl-(bim)(bimS)][Cl]**_**2**_, were not soluble in the reaction mixture under the experimental conditions used, and acted as heterogeneous catalysts. In general, yields increased by raising the amount of catalyst until reaching a limiting value. However, in all cases a catalyst loading of 20 mol % represented a good compromise between yields and concentration of acidic imidazolium salts. Accordingly, we carried out all reactions using this amount of catalyst. It could be quite surprising the fact that, both in the case of mono- and dicationic salts, the same amount of catalyst gave rise to the best performance. However, in our opinion, this could be ascribed to the action of factors different from reaction media acidity that operate on the studied process (see later).

From now on, for the sake of clarity, we will deal with the conversion of fructose and sucrose separately.

### Conversion of Fructose in 5-HMF

**Figure 5:**
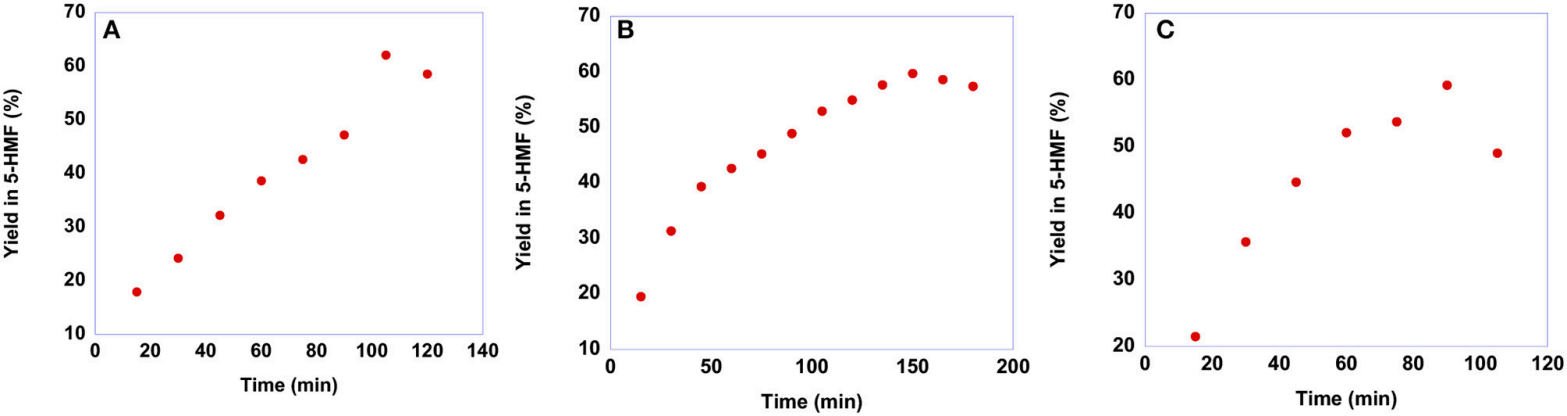
Plots of yields in 5-HMF obtained at 60 °C from fructose in the presence 20 mol % of **(A) [b**_**2**_**imS][Cl], (B) [*o*-Xyl-(bimS)**_**2**_**][Cl]**_**2**_ and **(C) [*m*-Xyl-(bimS)**_**2**_**][Cl]**_**2**_ as a function of time.

To gain information on the suitable reaction time, we carried out kinetic experiments monitoring the yield in 5-HMF as a function of time. It is worth noting that we did not perform kinetic investigations in the case of *p*-isomers as a consequence of their low solubility in the solvent mixture. Plots of yields in 5-HMF obtained from fructose as a function of time are reported in Figure 5, while yield values are reported in Table S4.

In general, the trends of yields as a function of time describe a curve, reaching a maximum yield at a given time and declining at longer reaction times. This drop in yield results from acid-catalyzed degradation processes of 5-HMF into humins or levulinic acid (Girisuta et al., 2006). Moreover, a closer inspection of the plots reported in Figure 5 reveals that the maximum yield in 5-HMF is the same, 60%, irrespective of the catalyst used. Conversely, the nature of the catalyst affects the rate of the process. Indeed, the time needed to reach the best yield increases along the order: **[*m*-Xyl-(bimS)**_**2**_**][Cl]**_**2**_<**[b**_**2**_**imS][Cl]**<**[*o*-Xyl-(bimS)**_**2**_**][Cl]**_**2**_ (t_max_ = 90, 105, 150 min for **[*m*-Xyl-(bimS)**_**2**_**][Cl]**_**2**_, **[b**_**2**_**imS][Cl]** and **[*o*-Xyl-(bimS)**_**2**_**][Cl]**_**2**_, respectively).

**Figure 6:**
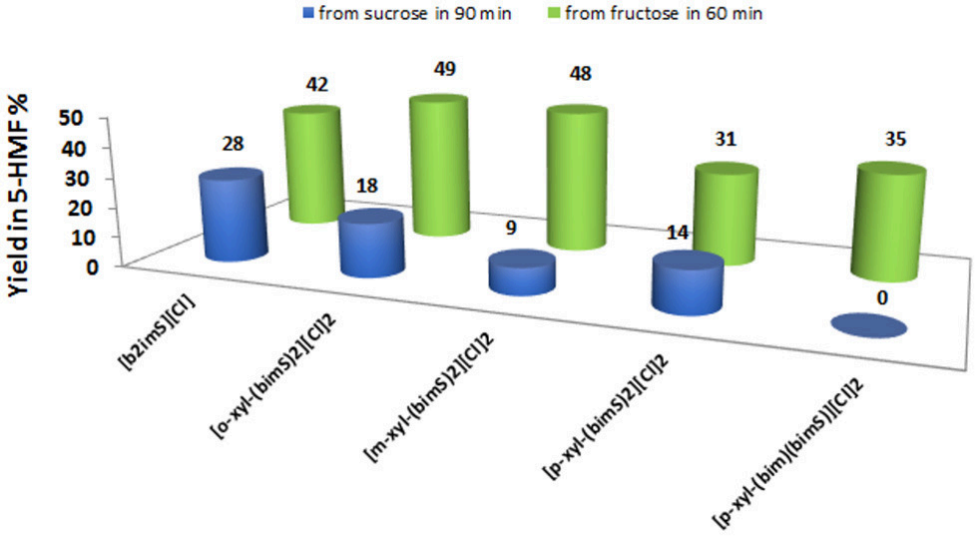
Yields in 5-HMF from fructose (after 60 min) and from sucrose (after 90 min) at 60°C, at 20 mol % of catalyst and in the IL binary mixture.

Anyway, to obtain a more meaningful comparison between homogeneous and heterogeneous catalysts, we chose to compare yields in 5-HMF obtained at the same time, 60 min, for all salts. For the sake of comparison, we performed the reactions also in ethanol and DMSO solution, using the **[*o*-Xyl-(bimS)**_**2**_**][Cl]**_**2**_ as catalyst, on the grounds of its good performance in the IL binary mixture. The results obtained in the IL binary mixture are reported in Figure 6.

The plot reported in Figure 6 reveals that the *p*-substituted salts **[*p*-Xyl-(bimS)**_**2**_**][Cl]**_**2**_ and **[*p*-Xyl-(bim)(bimS)][Cl]**_**2**_ are less efficient in enhancing the rate of the process compared with the other salts. Indeed, for dicationic salts, yields decrease along the following order: **[*o*-Xyl-(bimS)**_**2**_**][Cl]**_**2**_≈ **[*m*-Xyl-(bimS)**_**2**_**][Cl]**_**2**_> **[*p*-Xyl-(bimS)**_**2**_**][Cl]**_**2**_≈ **[*p*-Xyl-(bim)(bimS)][Cl]**_**2**_. The above result could be easily explained by considering that, in the presence of *p*-isomers, the reaction takes place in a heterogeneous system. Furthermore, the different symmetry or the different acidity (see later) of the [*p*-Xyl-(bim)(bimS)]^2+^ and [*p*-Xyl-(bimS)_2_]^2+^ cations does not appear to significantly affect the yield. Finally, considering the homogeneous catalysts, the dicationic salts give comparable yields with respect to monocationic one, suggesting that catalytic efficiency cannot be directly ascribed to the presence of two acidic functionalities.

As far as the comparison with conventional organic solvents is concerned, in DMSO solution we obtained a lower yield with respect to the IL binary mixture (39 and 49 % in DMSO and IL binary mixture, respectively). Conversely, in ethanol, we did not detect 5-HMF formation, probably as a consequence of the very low solubility of the catalyst in this solvent.

Given that the best result were obtained in the presence of **[*o*-Xyl-(bimS)**_**2**_**][Cl]_2_,**we tested the recyclability of the IL-based catalytic system. To this aim, we tested different extracting solvents and found that 5-HMF could be extracted using diethyl ether (4 × 20 mL). This amount of solvent is in line with what previously reported in the literature (Moreau et al., 2006) due to the known resistance of 5-HMF to be extracted from imidazolium-based ILs (Xiao and Huang, 2018). Using other solvents, like ethyl acetate, toluene, dimethyl carbonate or 2-methylpentanone resulted in insufficient recovery of 5-HMF or loss of IL in the organic phase. Finally, adding water in the reaction mixture prior to extraction had a detrimental effect on the yield in the subsequent cycle, which plummeted to 20%.

Using only diethyl ether, allowed us to extract 5-HMF in the organic phase while leaving intact the catalyst in the IL phase.

**Figure 7:**
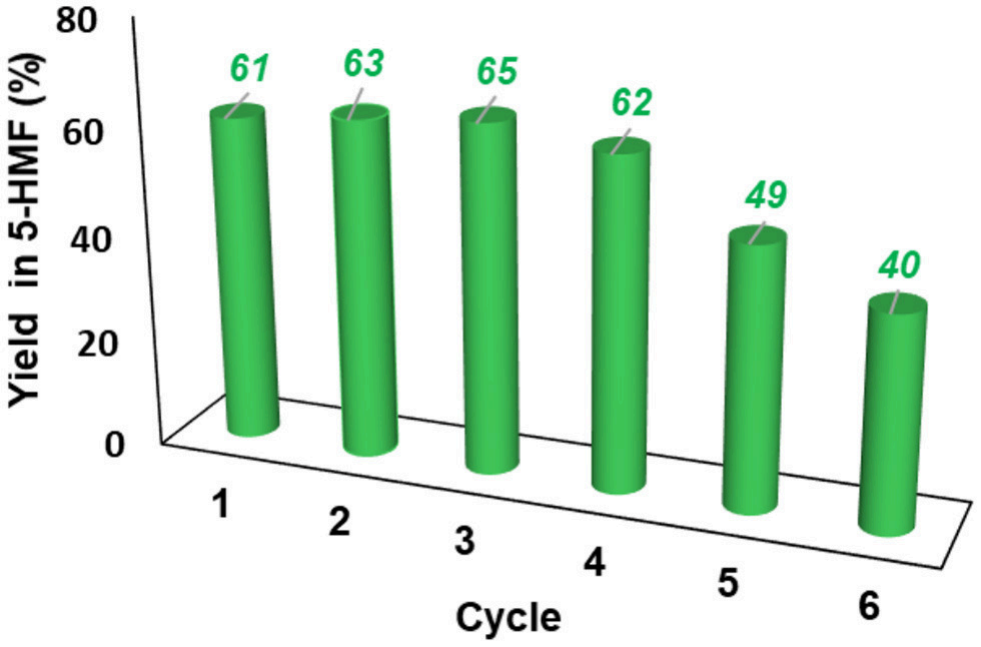
Yield in 5-HMF obtained at 60°C from fructose in the presence 20 mol % of **[*o*-Xyl-(bimS)**_**2**_**][Cl]**_**2**_ upon recycling of the catalytic system.

The resulting IL mixture was then added with a fresh batch of fructose and the reaction was carried out as already described. The results obtained are summarized in Figure 7.

Results reported in Figure 7 show that the IL mixture could be reused for 4 cycles without appreciable loss in yield. Even if further recycling led to a regular decrease in yield, the catalytic system could be still reused for two additional cycles obtaining good yields.

**Figure 8:**
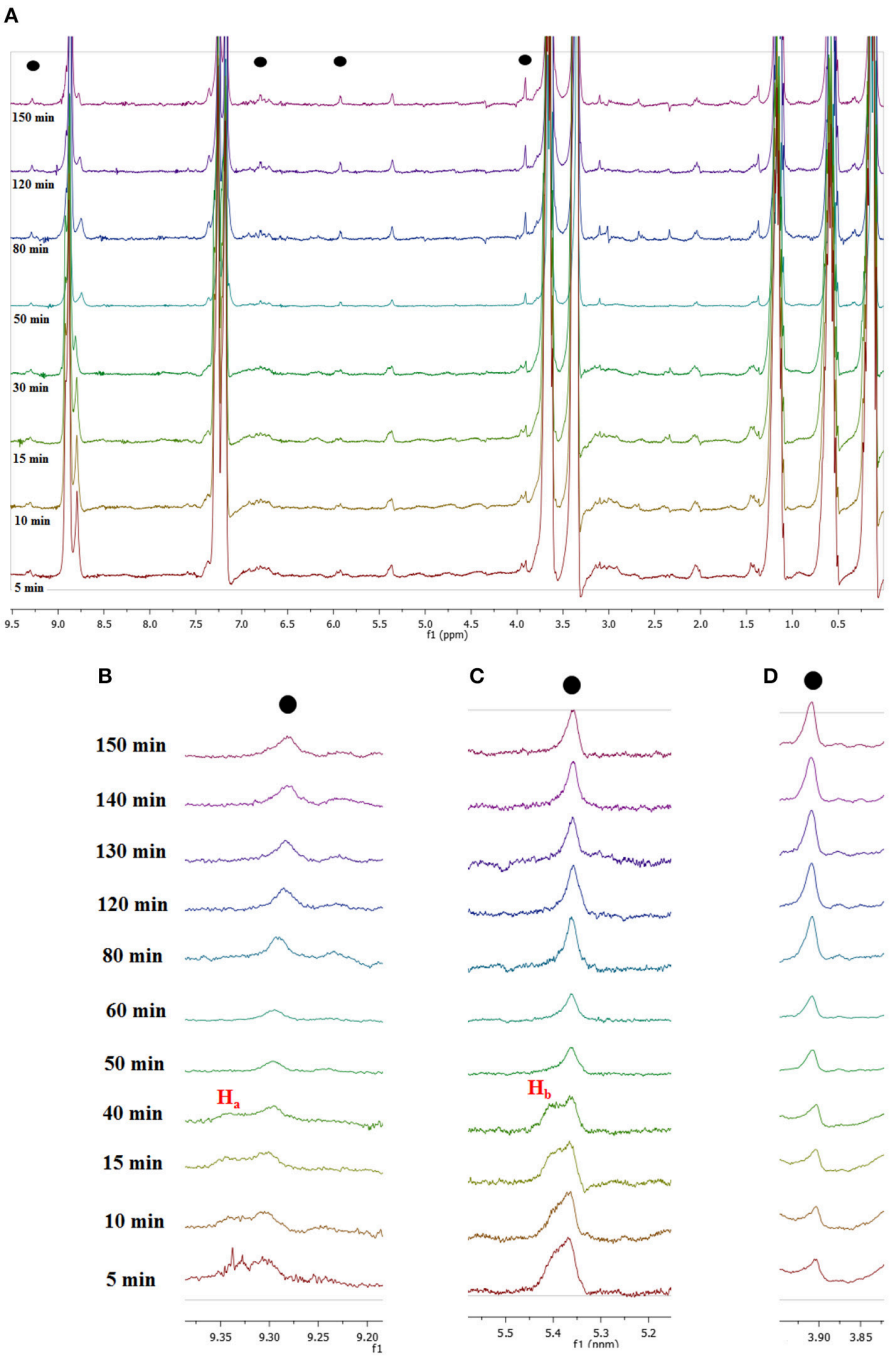
**(A)** ^1^H NMR spectra recorded for *in situ* conversion of fructose into 5-HMF at selected times and local enlargements of the chemical shifts region of **(B)** 9.20–9.40 ppm, **(C)** 5.20–5.50 ppm, and **(D)** 3.80–3.95 ppm.

To obtain mechanistic information, we carried out *in situ* ^1^H-NMR measurements monitoring the dehydration of fructose into 5-HMF in a NMR-tube in the presence of **[*o*-Xyl-(bimS)**_**2**_**][Cl]**_**2**_, under the same experimental conditions described. Magnified ^1^H-NMR spectra recorded at selected times and their enlargements are reported in Figures 8, **S3**.

**Figure 9:**
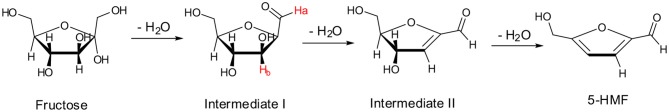
Intermediates hypothesized for the dehydration of fructose into 5-HMF.

A first glance at spectra reported in Figure 8A brings out the appearance and growth in intensity of the signals relevant to the protons of 5-HMF, labeled as (●). Furthermore, enlarging suitable regions of the spectra shows the presence of signals which gradually disappear with time and consequently can be attributed to the occurrence of intermediates. These can be observed at 9.35 ppm, (H_a_, Figure 8B) and 5.40 ppm (H_b_, Figure 8C). We explained these findings by hypothesizing the occurrence of a mechanism similar to that recently reported by Huang for the dehydration of fructose in a IL binary mixture, (Zhang et al., 2015; Xiao and Huang, 2018) featuring two cyclic intermediates (Figure 9).

In particular, the signal of the proton at 9.35 ppm which disappeared after 50 min, can be attributed to the aldehydic proton H_a_ of intermediate I. Analogously, in the region of chemical shifts between 5.20 and 5.50 ppm in the first minutes of the reaction a broad resonance, resulting from the overlap of two signals can be observed (Figure 8C). After 50 min, the signal centered at 5.40 ppm disappeared, living a single peak centered at 5.30 ppm. We explain this finding by attributing the signal at 5.40 ppm to the H_b_ proton of intermediate I. We observe the disappearance of these two signals, after the same time, 50 min. Their disappearance is therefore synchronous, which lends further support to the hypothesis that these signals belong to the same intermediate. Finally, in Figure 8D the appearance of the methylenic protons of 5-HMF can be observed at 3.95 ppm.

It is important to note that this is a simplified representation and that the full mechanistic picture can be much more complicated than that. However, a full mechanistic investigation is outside the scope of the present work.

Since the conversion of fructose into 5-HMF is an acid catalyzed process, we tried to understand the trend observed by measuring the acid strength of our sulfonic acid-functionalized imidazolium salts. To this aim, we assessed the acidity of our salts by determining the *H*_0_ acidity function, using the Hammett indicator method (Hammett and Deyrup, 1932; Thomazeau et al., 2003). This approach has been applied to determine the acidity of sulfonic acid-functionalized imidazolium-based ILs (Kore and Srivastava, 2012; Yaman et al., 2017). In particular, we determined the *H*_0_ function values for our salts using methyl orange as indicator in solution of methanol (p*K*_aMeOH_ = 3.8) (Kolthoff and Guss, 1938).

The choice of methanol was firstly due to the good solubility of all our catalysts in this solvent, differently from the case of water solution where *p*-isomers were not soluble at the concentration needed. Furthermore, as previously reported, polarity of imidazolium-based ILs is comparable to alcohols bearing shorter alkyl chains (Carmichael and Seddon, 2000).

We are aware that the acid strengths detected in methanol could be different than those occurring in solution of ILs. Nevertheless, this determination of the *H*_0_ function can still give a qualitative understanding of the different acidities of our salts and their ability to promote the reaction. To obtain a meaningful comparison among salts bearing one and two sulfonic acid groups, all determinations were carried out in solutions containing the same total proton concentration. Full details of calculations are given in the experimental section, while *H*_0_ values are reported in Table 1.

**Table 1:** *H*_0_ values for each salt determined at 298 K using methyl orange as indicator ^a^.

**Salt**	***H*_0_^b^**
[b_2_imS][Cl]	3.8
[*o*-Xyl-(bimS)_2_][Cl]_2_	3.9
[*m*-Xyl-(bimS)_2_][Cl]_2_	4.1
[*p*-Xyl-(bimS)_2_][Cl]_2_	4.0
[*p*-Xyl-(bim)(bimS)][Cl]_2_	5.9

a H_0_ values were reproducible within ± 0.1.

b *pK_a_ of methyl orange in MeOH = 3.80*.

The analysis of results reported in Table 1 reveals that our salts show comparable acidities. Furthermore, our dicationic TSILs exhibit acidity comparable to monocationic one **[b**_**2**_**imS][Cl]**. The only exception is represented by **[*p*-Xyl-(bim)(bimS)][Cl]**_**2**_, in which case a significantly lower acidity was detected.

However, trend in *H*_0_ values does not allow rationalizing the reactivity trend, indicating that although the acidity plays a role, it is not able to explain alone the experimental data. Such inability to find a simple correlation between Hammet acidity or basicity and reactivity data has been already reported in literature. On this subject, we found a similar behavior performing the mononuclear rearrangement of heterocycles in the presence of dicationic TSILs (Rizzo et al., 2014). Previously, Chen et al. studying the Beckmann rearrangement in the presence of some acidic dicationic ILs, obtained comparable yields in amides regardless of the acidic strength of ILs, as accounted for by *H*_0_ values (Liu et al., 2009). On the other hand, also for the dehydration of fructose, the trend in yield has been ascribed to the concomitant action of several factors on the transformation, different from the Hammett acidity (Dam et al., 1986).

Another parameter that can be affected by the relative position of the substituent in the phenyl ring is the dipole moment of the cation. In particular, for related dicationic isomeric salts, Yaman et al. reported that the dipole moment increases in the order para < meta < orto and that impacts on the acidity of the protons on the imidazolium rings (Yaman et al., 2017). However, in our case, this contribution is negligible, and the acidity of the isomeric dicationic salts is practically the same, due to the overwhelming effect of the strongly acidic -SO_3_H function. For this reason, in our case, dipole moment does not play a significant role in explaining the reactivity observed.

Conversely, taking in consideration the catalysts soluble in the binary mixture of ILs, the concomitant action of cation structure and acidity could explain the reactivity trend. Indeed, the significant difference detected in the t_max_ values measured for **[*m*-Xyl-(bimS)**_**2**_**][Cl]**_**2**_and **[*o*-Xyl-(bimS)**_**2**_**][Cl]**_**2**_ could be ascribed to the increased proximity of imidazolium heads on the *o*-xylilene spacer. This, could induce two different effects that decrease the catalytic ability of the corresponding TSIL. On the one hand, after the first deprotonation event, the second proton could be involved in hydrogen bonding interaction with the sulfonate group which, in turn, makes less probable its release in solution. On the other hand, the proximity of imidazolium heads could slow down the second deprotonation event, as a consequence of the electrostatic repulsion between sulfonate groups.

In this regard, it has been previously reported that for similar diimidazolium salts the isomeric substitution affects the conformations of cations and properties of the relevant IL (D'Anna et al., 2009a). Unfortunately, solubility issues do not allow drawing similar conclusions in the case of *p*-isomers.

In general, our task specific dicationic ILs show better performance in fructose conversion with respect to the ones previously reported by Jadhav et al. (2012) Indeed, in that case, yield values ranged from 70 up to 90 %, but using an equimolar amount of catalyst at 120°C.

### Conversion of Sucrose in 5-HMF

**Figure 10:**
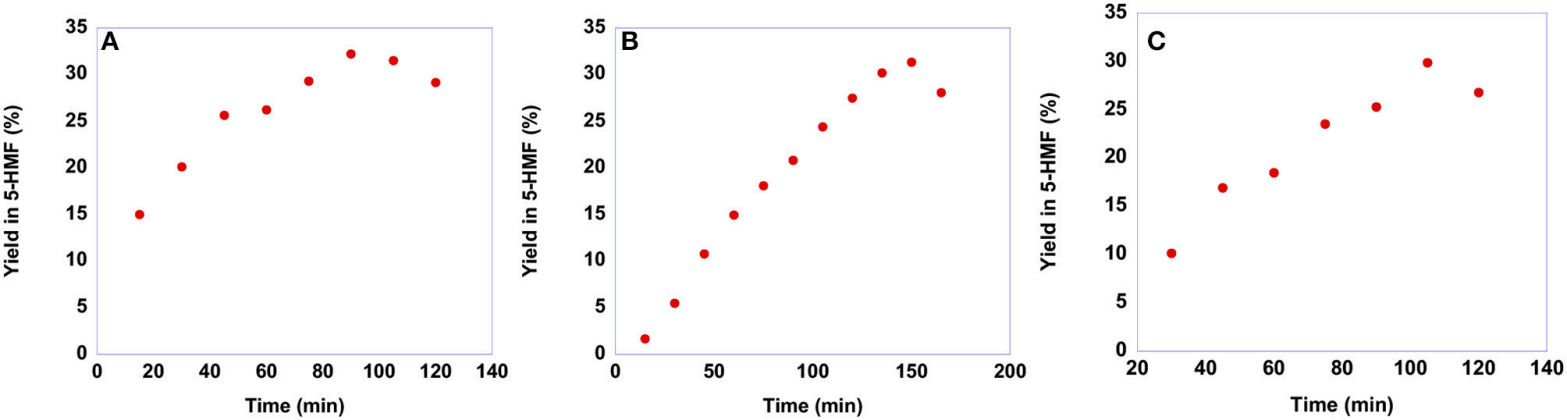
Plots of yields in 5-HMF obtained at 60°C from sucrose in the presence 20 mol % of **(A) [b**_**2**_**imS][Cl]**, **(B) [*o*-Xyl-(bimS)**_**2**_**][Cl]**_**2**_ and **(C) [*m*-Xyl-(bimS)**_**2**_**][Cl]**_**2**_ as a function of time.

As already said, the conversion of sucrose into 5-HMF is a more difficult process compared with that of fructose. Consequently, sucrose conversion often shows lower yields in 5-HMF and requires harsher conditions. To assess whether our salts are suitable for obtaining 5-HMF from sucrose, we conducted the reactions at 60°C, the same temperature used for the conversion of fructose, maintaining the same reaction conditions also in terms of catalyst loading, 20 mol %, with respect to the substrate. In particular, also in this case we monitored the yield in 5-HMF as a function of time. Plots of yields in 5-HMF obtained from sucrose as a function of time are reported in Figure 10, while the yield values are reported in Table S5.

Looking at the plots reported in Figure 10 reveals that even at this relatively low temperature, a yield of 30% is obtained after 90 min in the case of **[b**_**2**_**imS][Cl]**, 105 min in the case of **[*m*-Xyl-(bimS)**_**2**_**][Cl]**_**2**_ and 135 min in the presence of **[*o*-Xyl-(bimS)**_**2**_**][Cl]**_**2**_. These findings represent a marked improvement compared with our previous investigation of sucrose conversion in 5-HMF, in ILs, catalyzed by Amberlyst 15. In that case a lower yield, 23% was obtained after 120 min but required a much higher temperature, 90°C (D'Anna et al., 2014). Furthermore, notwithstanding in two cases the catalysts bear the same acidic functions, the TSILs used in this paper allowed us to operate under less acidic conditions compared with the analogous reaction carried out in the same ILs mixture in the presence of Amberlyst 15 (2.35·10^−4^ mol H^+^ and 5.55·10^−5^ mol H^+^ for Amberlyst 15 and dicationic TSILs, respectively) (D'Anna et al., 2014).

Notably, a comparable yield has been reported in literature for the obtainment of 5-HMF from sucrose in the presence of FePO_4_ in THF/H_2_O after 1h at 140°C (Yang et al., 2015). Some reports in the literature show the obtainment of higher yields in 5-HMF from sucrose (Tian et al., 2013; Qu et al., 2016; Yu et al., 2017). However, in all these cases either metal catalysts or very high temperatures and thus harsh reaction conditions are required. Consequently, we claim that the much milder reaction conditions and the absence of toxic metal catalyst make the use of our salts convenient from the standpoint of green chemistry.

Also in this case, we compared the rate of sucrose conversion in 5-HMF in the presence of all catalysts in the IL binary mixture and in ethanol and DMSO solution, using the **[*o*-Xyl-(bimS)**_**2**_**][Cl]**_**2**_ as catalyst, by considering the yield in 5-HMF obtained at the same time, 90 min. The results, collected in the IL binary mixture, are reported in Figure 6.

These findings show that yields in 5-HMF display a more articulate trend compared with the one observed in the case of fructose conversion. In particular, while the monocationic salt **[b**_**2**_**imS][Cl]** affords the fastest conversion, no 5-HMF is formed after 90 min in the presence of the non-symmetrically substituted salt **[*p*-Xyl-(bim)(bimS)][Cl]**_**2**_. Furthermore, yields increase by following the order: **[*m*-Xyl-(bimS)**_**2**_**][Cl]**_**2**_<**[*p*-Xyl-(bimS)**_**2**_**][Cl]**_2_<**[*o*-Xyl-(bimS)**_**2**_**][Cl]**_**2**_<**[b**_**2**_**imS][Cl]**.

In the case of sucrose, the yield obtained in DMSO solution was comparable to the one obtained in the IL binary mixture.

Analogously to what happens in the case of fructose conversion, the trend obtained is not related to the acidity of the salts, as expressed by the *H*_0_ function. However, in this case the solubility of the salts in the reacting mixture does not prove a limiting factor and no single parameter is able to account for the trend observed. This can be the result of the concomitant action of several factors on different mechanistic steps featuring this complex pathway.

As previously stated the use of dicationic organic salts to obtain 5-HMF from carbohydrates has been scarcely explored, above all as far as sucrose is taken in consideration as starting material. Consequently, at this point, comparison with data available in literature is mandatory (Table 2).

**Table 2:** Reaction conditions and yield values for fructose and sucrose conversion in 5-HMF in the presence of task specific dicationic ILs.

**Catalyst**	**Fructose**		**Sucrose**		
	**Reaction temperature (°C)/Reaction time (min)**	**Yield (%)**	**Reaction temperature (°C)/Reaction time (min)**	**Yield (%)**	**References**
[DiEG(mim)_2_][OMs]_2_	120/40	70	120/150	54	Jadhav et al., 2012
[TriEG(mim)_2_][OMs]_2_	120/40	77		61	
[TetraEG(mim)_2_][OMs]_2_	120/40	92		67	
A-ortho Cl	100/60	50			Yaman et al., 2017
B-para Cl	100/60	<40			
C-meta Cl	100/60	<50			
A-ortho HSO_4_	100/60	90			
B-meta HSO_4_	100/60	60			
B-para HSO_4_	100/60	90			
[tetra(EG)(mim)trimethylamine][CH_3_SO_3_]	70/40	62			Jadhav et al., 2014
[tetra(EG) (mim)trimethylamo][Br]_2_	70/40	57			
[tetra(EG)(mim) trimethylamo][HSO_4_]_2_	70/40	92			
[*m*-Xyl-(bimS)_2_][Cl]_2_	60/90	60	60/105	30	This work
[*o*-Xyl-(bimS)_2_][Cl]_2_	60/150	60	60/135	30	This work

It is worth noting that, with only one exception in which the catalyst is used in DMSO solution, (Yaman et al., 2017) in the other cases reported in literature, task specific dicationic ILs are used both as solvent and catalyst; hence in a significantly higher amount. Bearing in mind this information, analysis of data reported in Table 2 underlines how, in the case of fructose, the advantage in using our catalysts derives from the lower reaction temperature. Indeed, with the only exception of **[tetra(EG)(mim)trimethylamo][HSO**_**4**_**]**_**2**_, that gives significantly higher yield in 5-HMF at a slightly higher temperature, in all the other cases comparable yields were obtained, using a significantly higher reaction temperature.

On the other hand, in the case of sucrose, yields ranging from 54 up to 67% were reported, but working at 120°C for 150 min.

## Conclusions

The study of fructose and sucrose conversion catalyzed by acid functionalized imidazolium salts in the [bmim][Cl]_0.5_[BF_4_]_0.5_ IL mixture, demonstrated that our salts are effective catalysts for the obtainment of 5-HMF in very mild conditions. Indeed, working at 60°C and using 20 mol % of catalyst, we obtained 60 and 30 % of 5-HMF from fructose and sucrose, respectively. The reaction medium and catalyst could also be successfully recycled for 4 times without loss of yield and for further two cycles obtaining lower but still good yields. Furthermore, by *in situ* ^1^H NMR monitoring of the reaction we hyphothesized the occurrence of a mechanistic pathway involving two intermediates.

The different substitution of the dicationic salts appeared to impact mainly the rate of the process and only marginally the overall yields. In particular, in the case of fructose the dicationic salts **[*m*-Xyl-(bimS)**_**2**_**][Cl]**_**2**_ and **[*o*-Xyl-(bimS)**_**2**_**][Cl]**_**2**_ lead to faster and slower reaction compared with the monocationic **[b**_**2**_**imS][Cl]**. Differently, in the case of sucrose, **[b**_**2**_**imS][Cl]** proved the best catalyst.

Evaluation of the acidity of the salts revealed almost identical acid strength for all the salts, with the exception of **[*p*-Xyl-(bim)(bimS)][Cl]**_**2.**_Finally, we identified the lower solubility of the *p*-substituted salts **[*p*-Xyl-(bimS)**_**2**_**][Cl]**_**2**_and **[*p*-Xyl-(bim)(bimS)][Cl]**_**2**_ as the reason for their poor catalytic performance in both cases.

On the whole, our results show that acidic TSILs do not behave as simple catalysts. Indeed, their performance cannot be attributed only to the amount of protons borne in the structure, but rather to the action of structural and conformational effects. Rather interestingly, in the ILs binary mixture used, our TSILs prove to be more efficient than some of previously reported catalytic systems both in terms of catalyst loading and reaction temperature, paving the way to more sustainable processes for the valorisation of raw materials.

For fructose conversion, the comparison with conventional organic solvents sheds light on a better efficiency of the tested catalyst, the **[*o*-Xyl-(bimS)**_**2**_**][Cl]**_**2**_, in the IL binary mixture with respect to DMSO solution. Probably, the increased efficiency can be ascribed to the different nature of the ion pair, representing the catalyst, in the two solvent systems, i.e., intimate and solvated ion pair in IL binary mixture and DMSO solution, respectively. Only in the first case, the TSIL could behave as a “bifunctional catalyst” that favors the carbohydrate dehydration both coordinating the hydroxyl group through hydrogen bond and favoring the water molecule departure as a consequence of the proton transfer.

## Author Contributions

All authors listed have made a substantial, direct and intellectual contribution to the work, and approved it for publication.

### Conflict of Interest Statement

The authors declare that the research was conducted in the absence of any commercial or financial relationships that could be construed as a potential conflict of interest.
