# Supplementary material for: Task-Specific Organic Salts and Ionic Liquids Binary Mixtures: A Combination to Obtain 5-Hydroxymethylfurfural From Carbohydrates
Source: Front Chem. 2019 Mar 21;7:134. doi: 10.3389/fchem.2019.00134 (PMC6437106; doi:10.3389/fchem.2019.00134)
Supplement: Supplementary file 1 [file Data_Sheet_1.PDF]

Supplementary Material

Task-specific Organic Salts and Ionic Liquids Binary mixtures: a Combination to Obtain 5-hydroxymethylfurfural from Carbohydrates

Salvatore Marullo, Carla Rizzo and Francesca D'Anna*

Università degli Studi di Palermo, Dipartimento di Scienze e Tecnologie Biologiche, Chimiche e Farmaceutiche, Viale delle Scienze, Ed. 17, Italia

*** Correspondence:** Prof. Francesca D'Anna: francesca.danna@unipa.it

Table of contents

Figure S1. Plots of yields in 5-HMF as a function of the amount of catalyst for fructose after 105 minutes and 60 °C, for sucrose at 150 minutes and 60 °C. **2**

Figure S2. ^{1}H and ^{13}C NMR spectra of the TSILs. **3**

Figure S3. Stacked magnified ^{1}H NMR spectra f situ for NMR analysis **8**

Figure S4. ESI Mass spectra of the TSILs **9**

Table S1. Yields in 5-HMF from fructose and sucrose at 60°C with and without performing hydrolysis as a function of **[b$_2$imS][Cl]** amount **12**

Table S2. Yields in 5-HMF from fructose, at 60 °C and after 105 minutes, as a function of catalyst amount. **13**

Table S3. Yields in 5-HMF from sucrose, at 60 °C and after 150 minutes, as a function of catalyst amount. **14**

Table S4. Yields in 5-HMF from fructose at 60°C, in the presence of 20 mol % of catalyst, as a function of time. **15**

Table S5. Yields in 5-HMF from sucrose at 60°C, in the presence of 20 mol % of catalyst, as a function of time. **16**

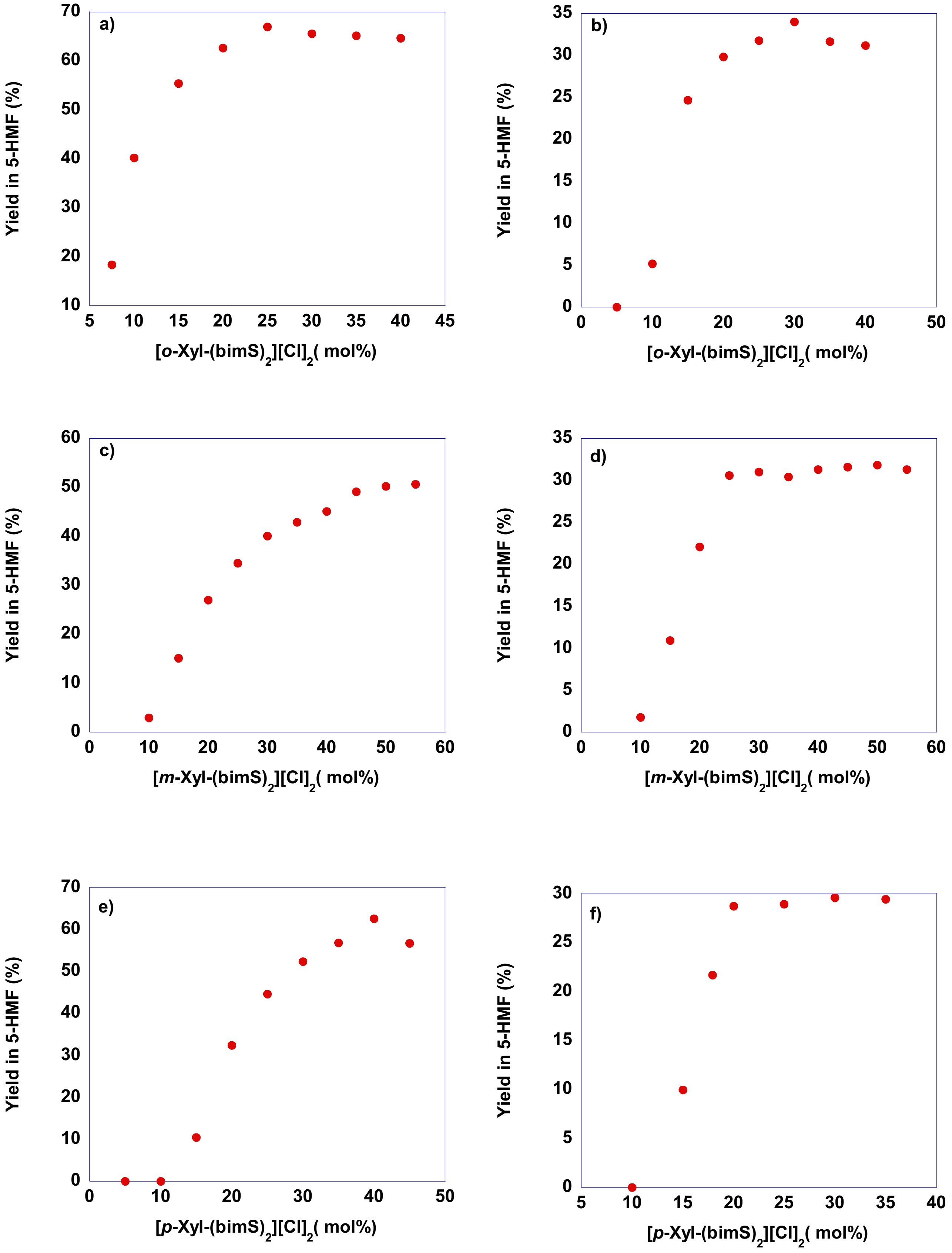
a)
Yield in 5-HMF (%)
[o-Xyl-(bimS)2][Cl]2(mol%)
b)
Yield in 5-HMF (%)
[o-Xyl-(bimS)2][Cl]2(mol%)
c)
Yield in 5-HMF (%)
[m-Xyl-(bimS)2][Cl]2(mol%)
d)
Yield in 5-HMF (%)
[m-Xyl-(bimS)2][Cl]2(mol%)
e)
Yield in 5-HMF (%)
[p-Xyl-(bimS)2][Cl]2(mol%)
f)
Yield in 5-HMF (%)
[p-Xyl-(bimS)2][Cl]2(mol%)

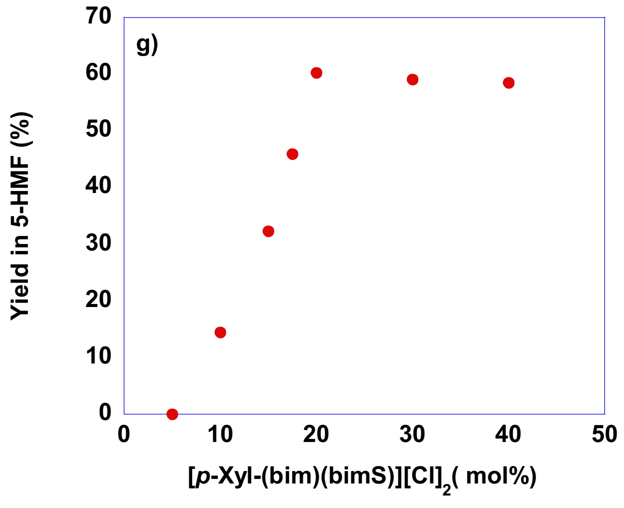

Figure S1. Plots of yields in 5-HMF as a function of the amount of catalyst: a), c), e), g) from fructose after 105 minutes and at 60 °C; b), d), f) sucrose after 150 minutes at 60 °C.

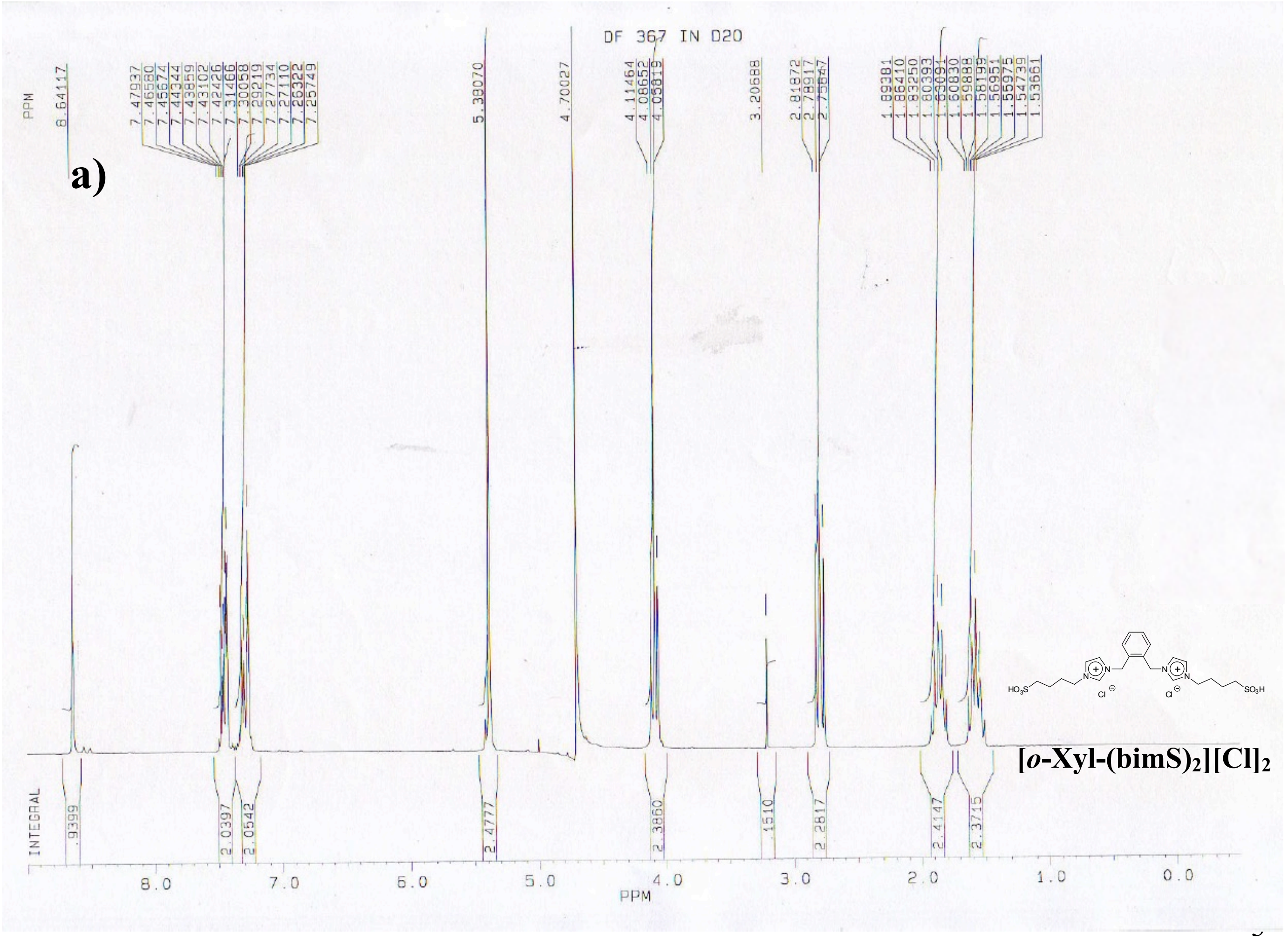

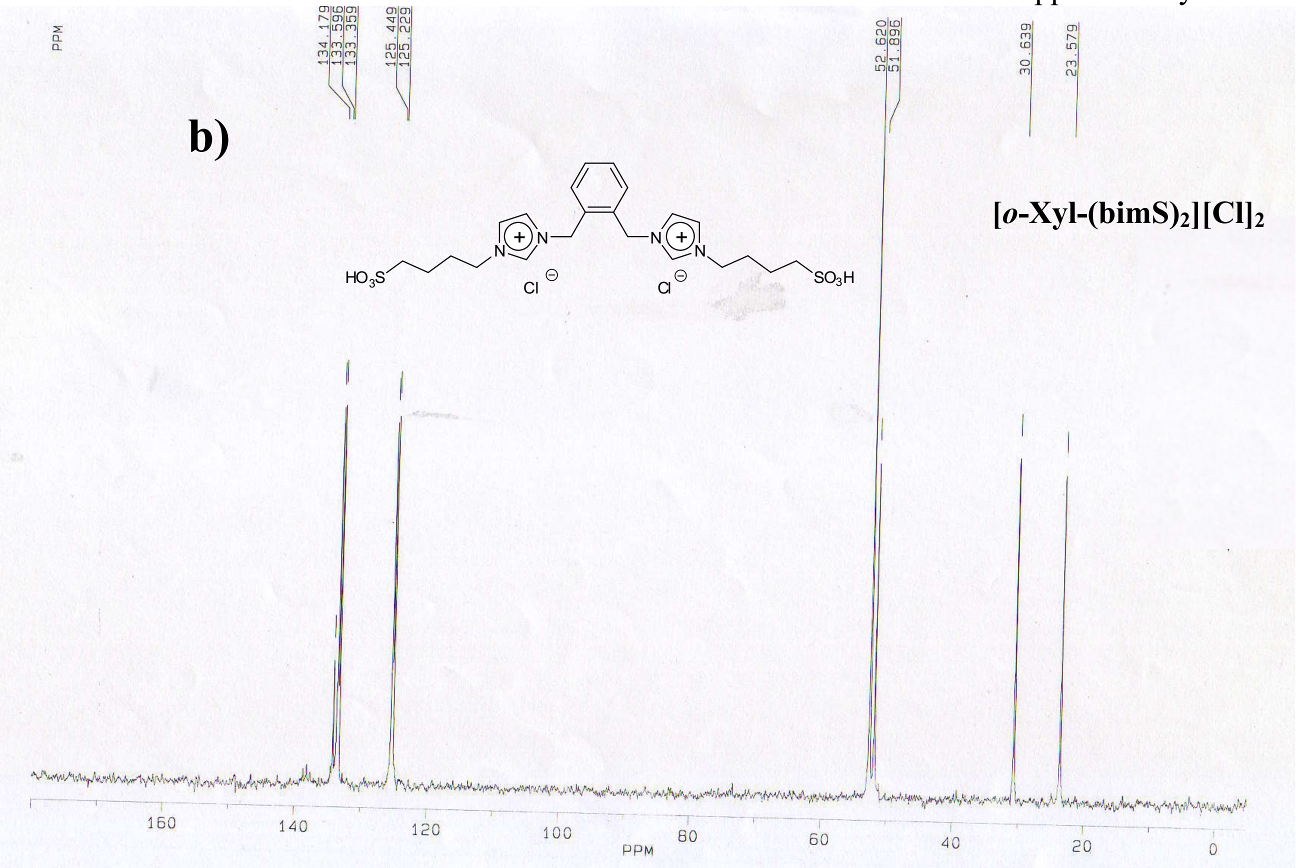
b)
[o-Xyl-(bimS)2][Cl]2
PPM

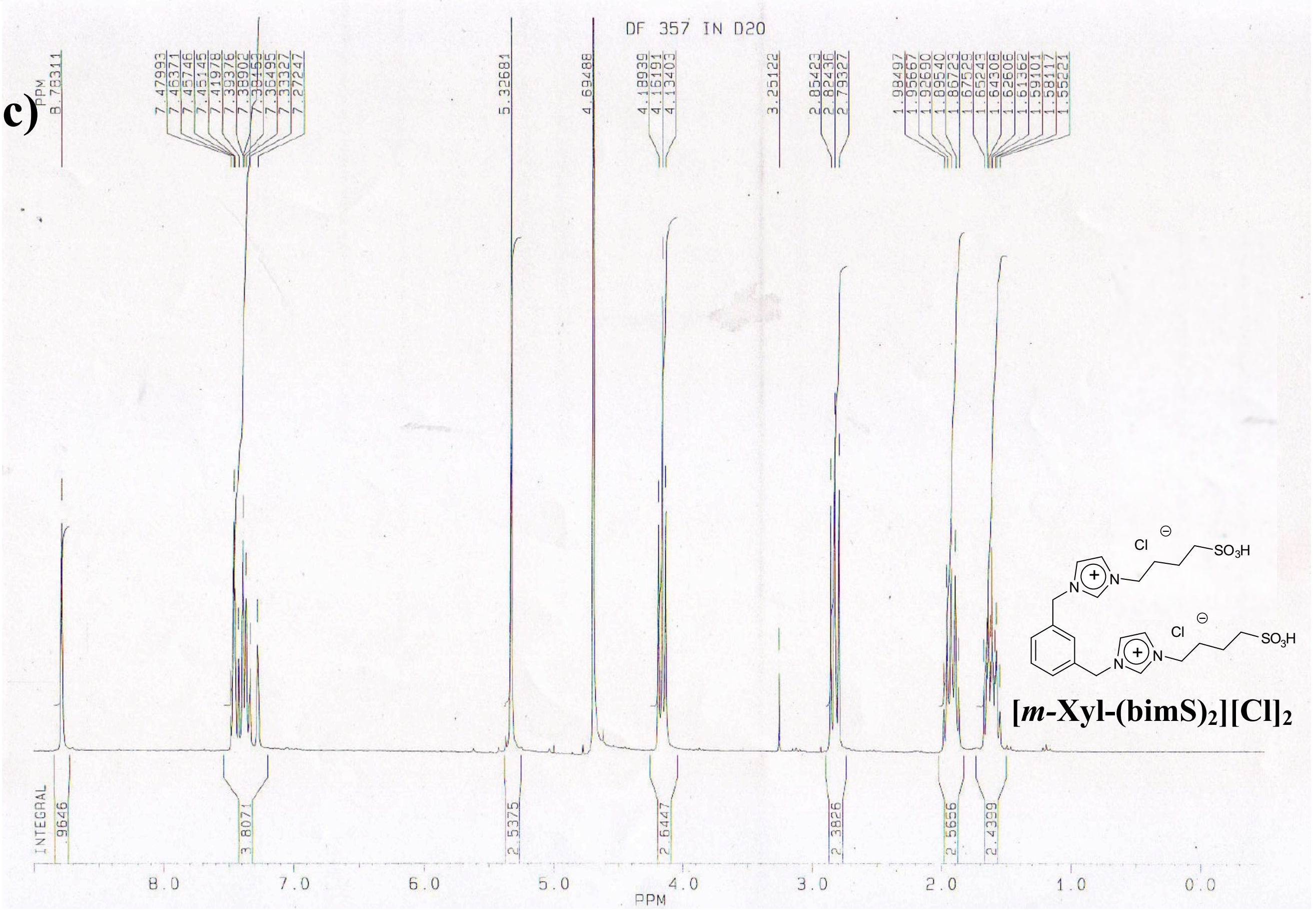
c)
DF 357 IN D2O
[m-Xyl-(bimS)2][Cl]2
INTEGRAL
PPM

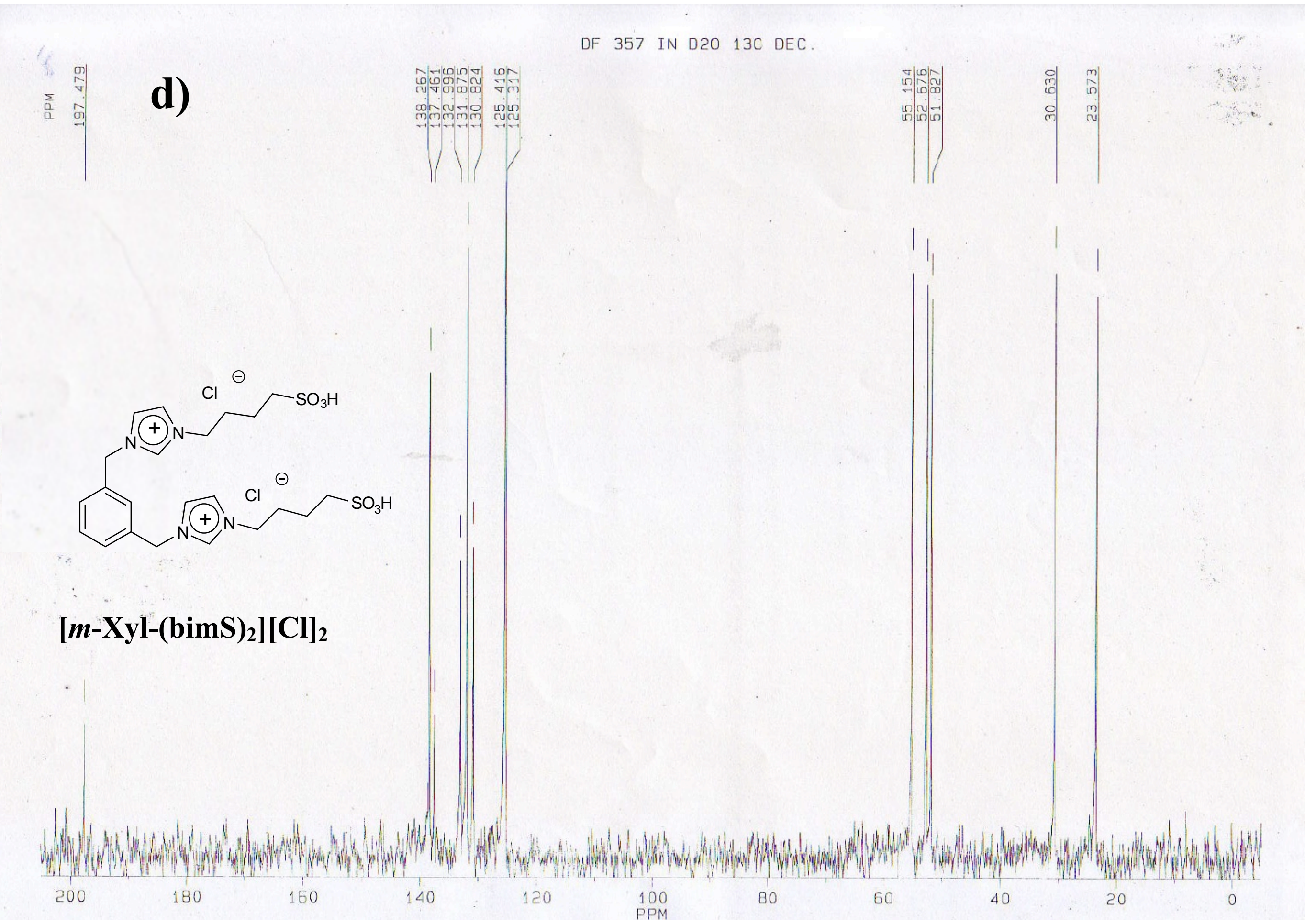
d)
DF 357 IN D2O 13C DEC.
PPM
197.479
138.267
137.461
132.996
131.875
130.824
125.416
125.317
55.154
52.576
51.827
30.630
23.573
Cl
SO3H
N
N
+
[m-Xyl-(bimS)2][Cl]2

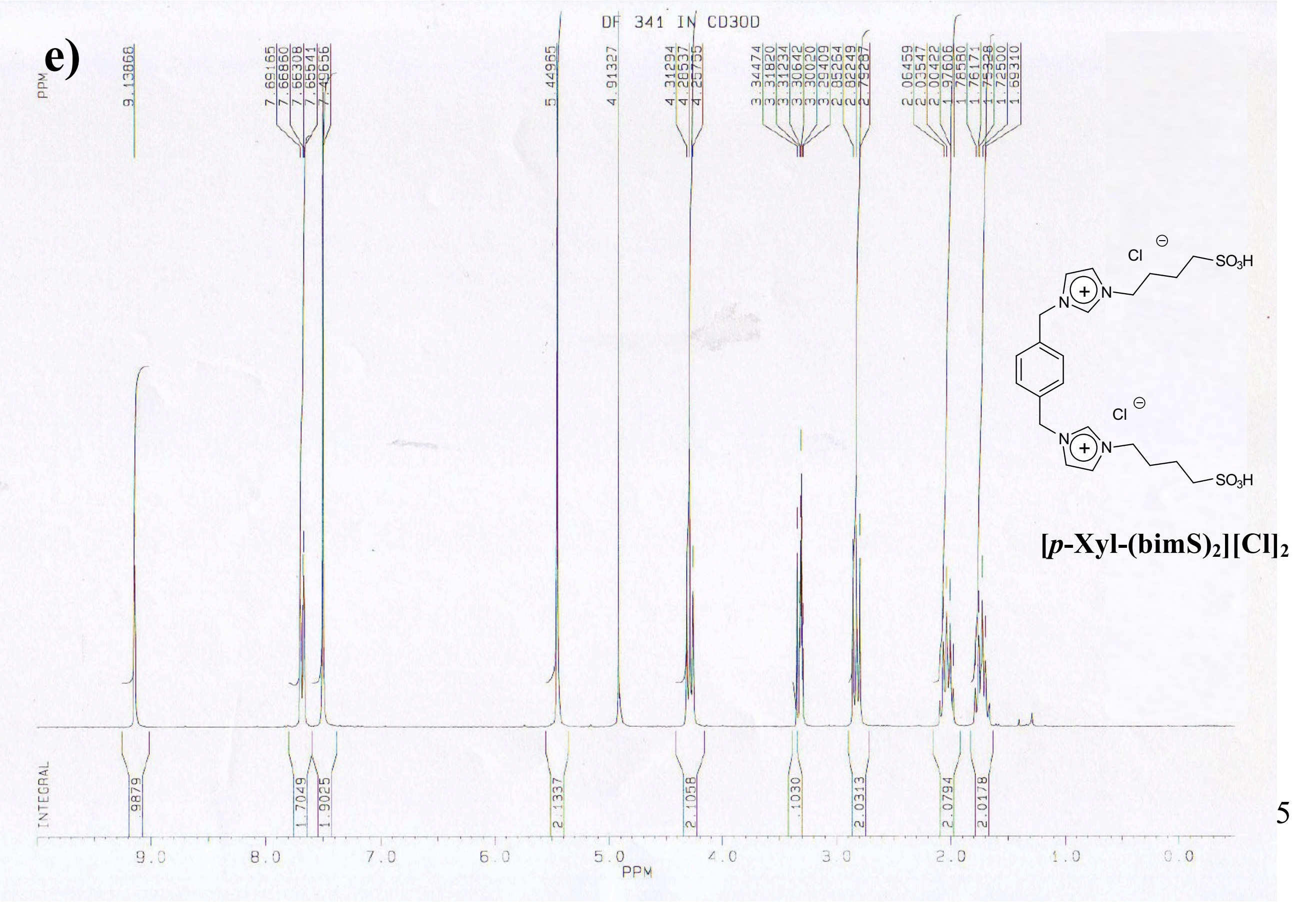
e)
DF 341 IN CD3OD
PPM
9.13866
5.44865
4.91327
4.31294
4.28537
4.25755
INTEGRAL
.9879
1.7049
1.9025
2.1337
2.1058
.1030
2.0313
2.0794
2.0178
Cl
SO3H
N
N
+
[p-Xyl-(bimS)2][Cl]2

f)

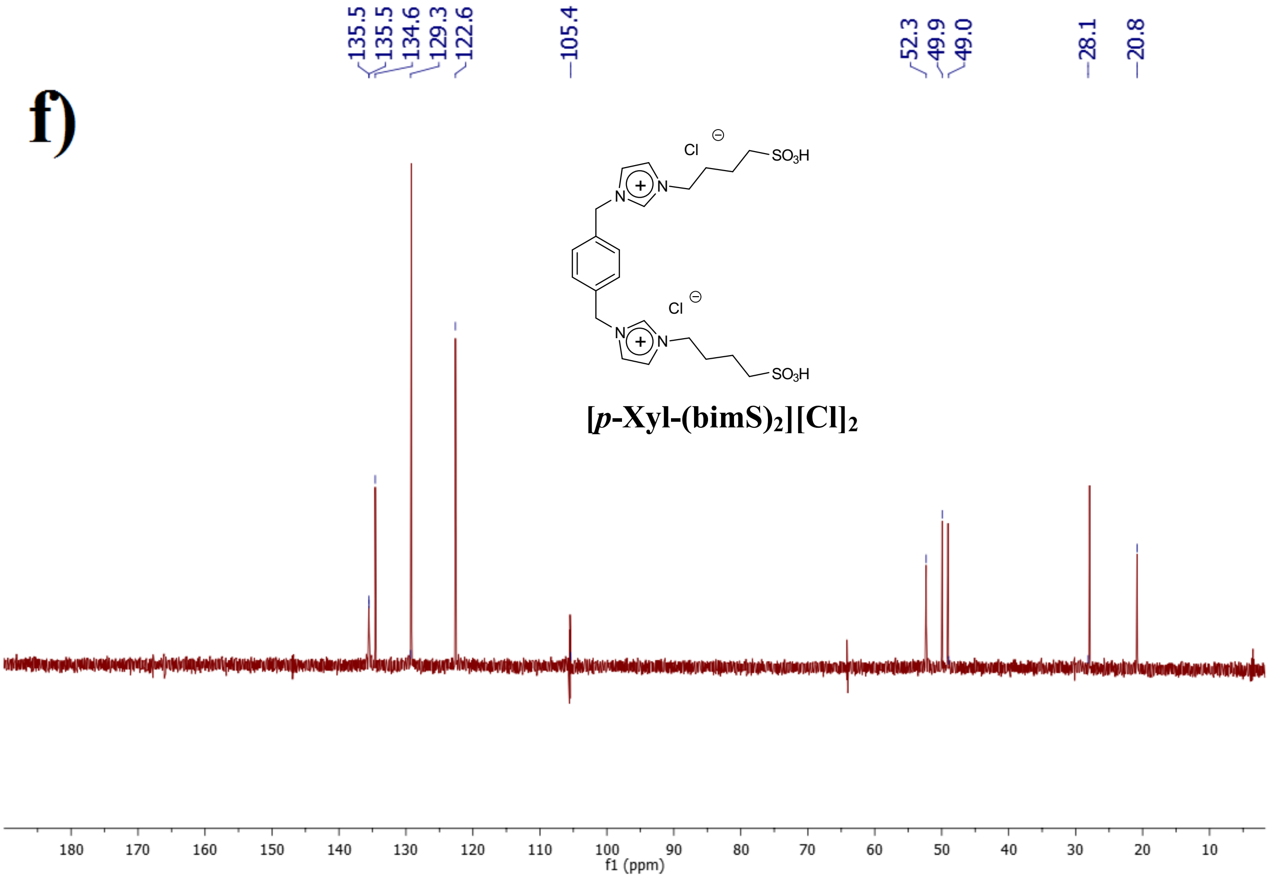
135.5
135.5
134.6
129.3
122.6
105.4
52.3
49.9
49.0
28.1
20.8
Cl
SO3H
N
N
[p-Xyl-(bimS)2][Cl]2
180
170
160
150
140
130
120
110
100
90
80
70
60
50
40
30
20
10
f1 (ppm)

g)

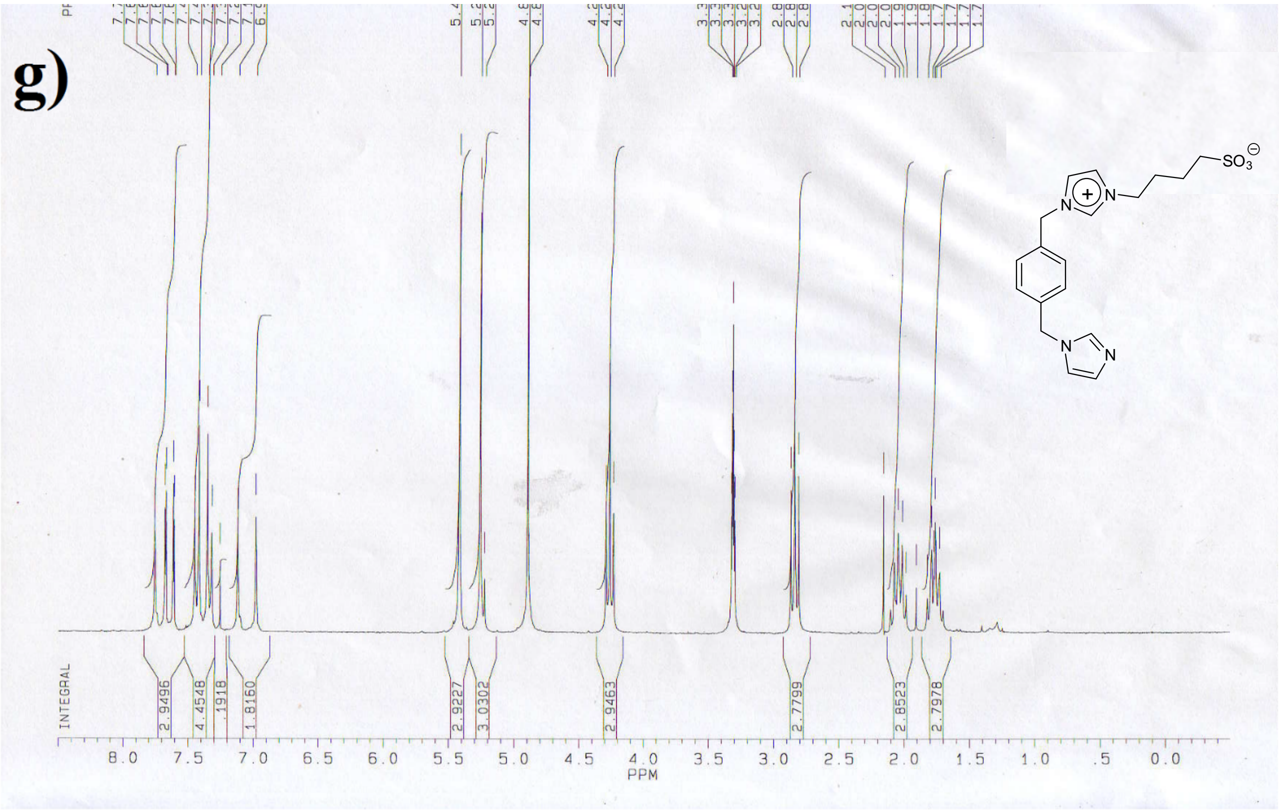
SO3
N
N
INTEGRAL
2.9496
4.4548
1918
1.8150
2.9227
3.0302
2.9463
2.7799
2.8523
2.7978
8.0
7.5
7.0
6.5
6.0
5.5
5.0
4.5
4.0
3.5
3.0
2.5
2.0
1.5
1.0
.5
0.0
PPM

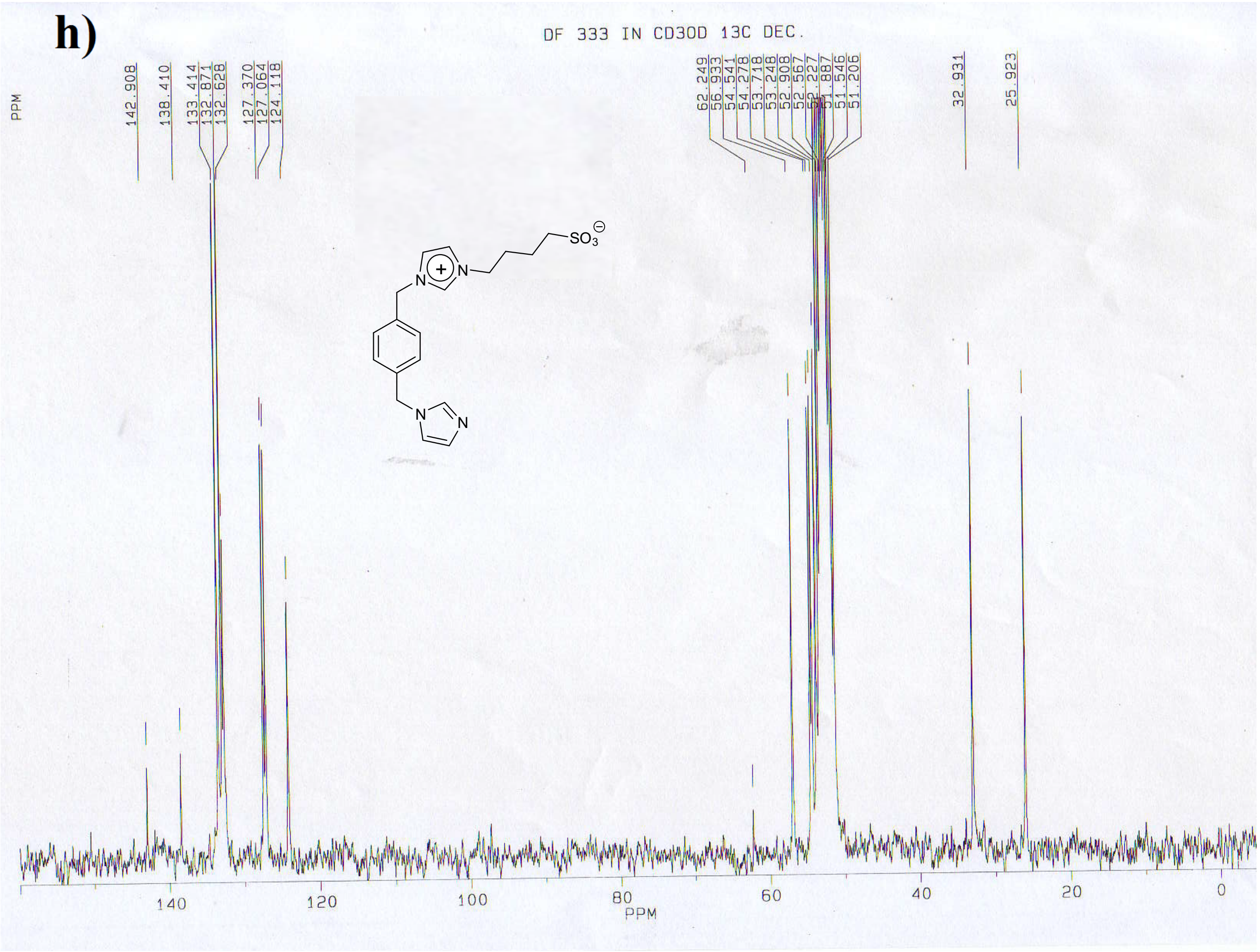
h)
DF 333 IN CD3OD 13C DEC.
PPM
142.908
138.410
133.414
132.871
132.628
127.370
127.064
124.118
62.249
56.933
54.541
54.278
53.718
53.248
52.908
52.567
52.227
51.887
51.546
51.206
32.931
25.923
140
120
100
80
60
40
20
0
PPM

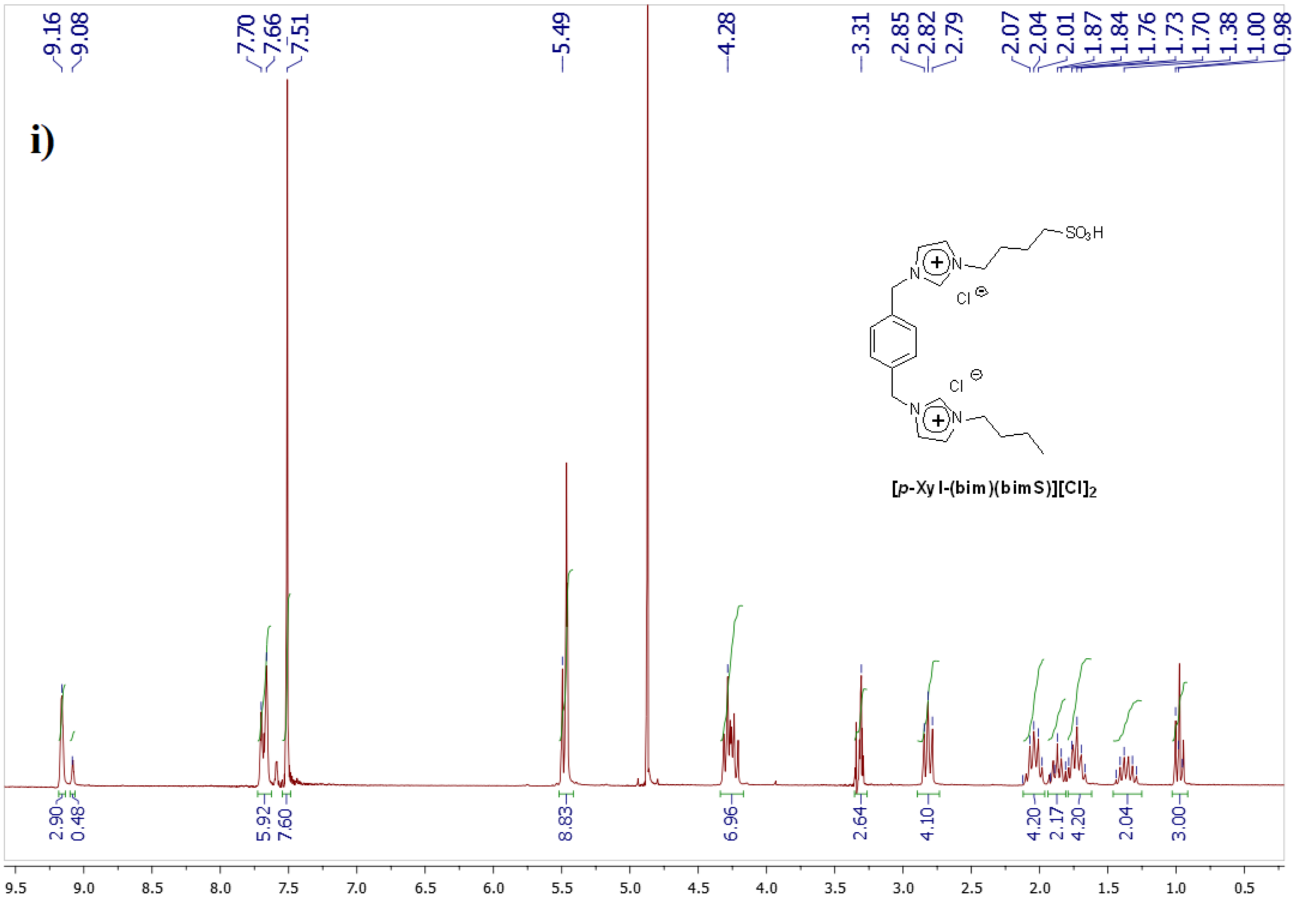
i)
9.16
9.08
7.70
7.66
7.51
5.49
4.28
3.31
2.85
2.82
2.79
2.07
2.04
2.01
1.87
1.84
1.76
1.73
1.70
1.38
1.00
0.98
2.90
0.48
5.92
7.60
8.83
6.96
2.64
4.10
4.20
2.17
4.20
2.04
3.00
[p-Xyl-(bim)(bimS)][Cl]2
9.5
9.0
8.5
8.0
7.5
7.0
6.5
6.0
5.5
5.0
4.5
4.0
3.5
3.0
2.5
2.0
1.5
1.0
0.5

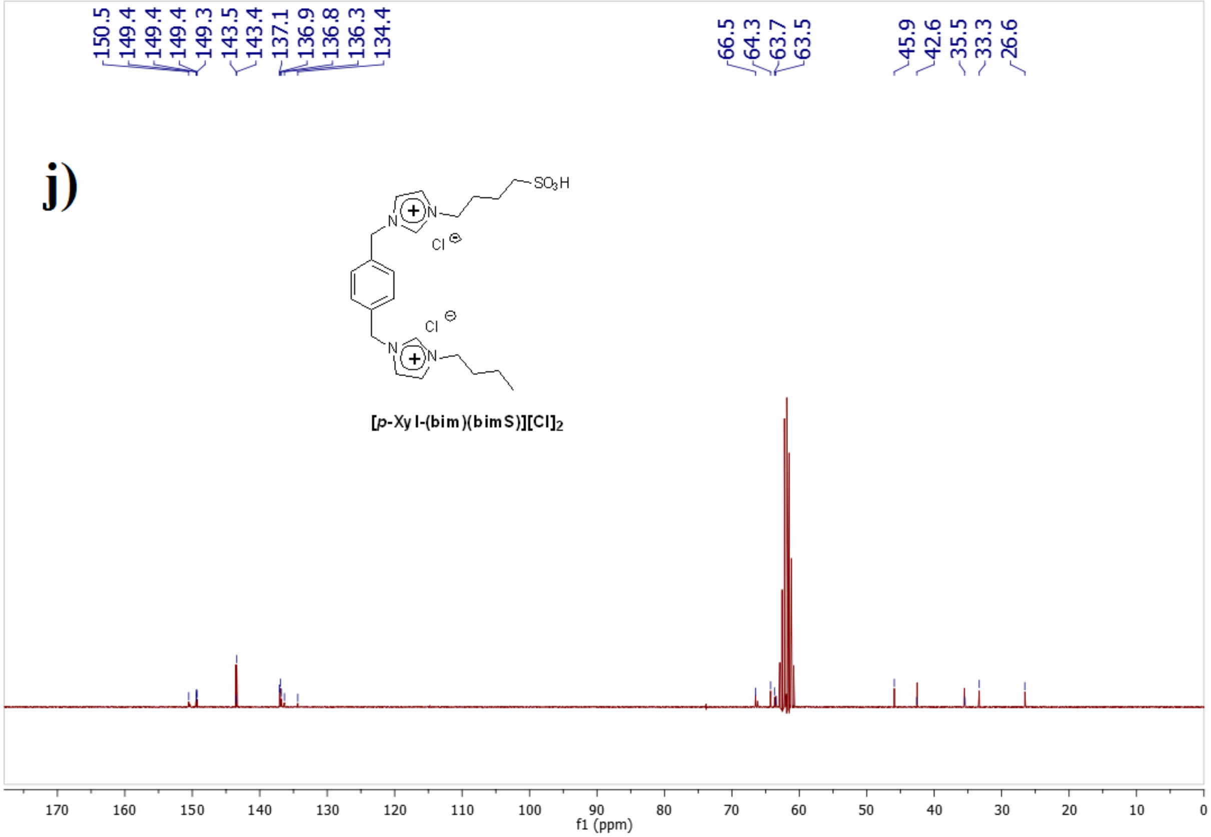

Figure S2. ^{1}H and ^{13}C NMR spectra of the TSILs.

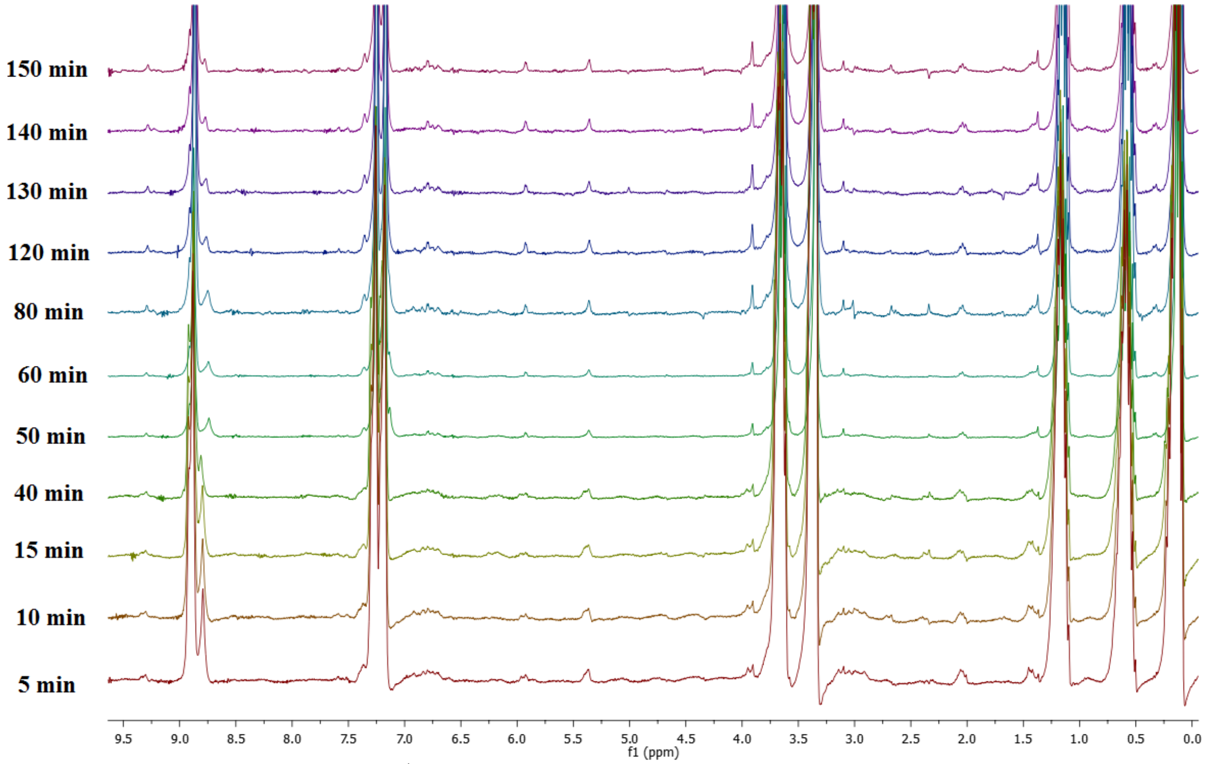

Figure S3. Stacked magnified ^{1}H NMR spectra relevant to in situ for NMR analysis for the dehydration of fructose.

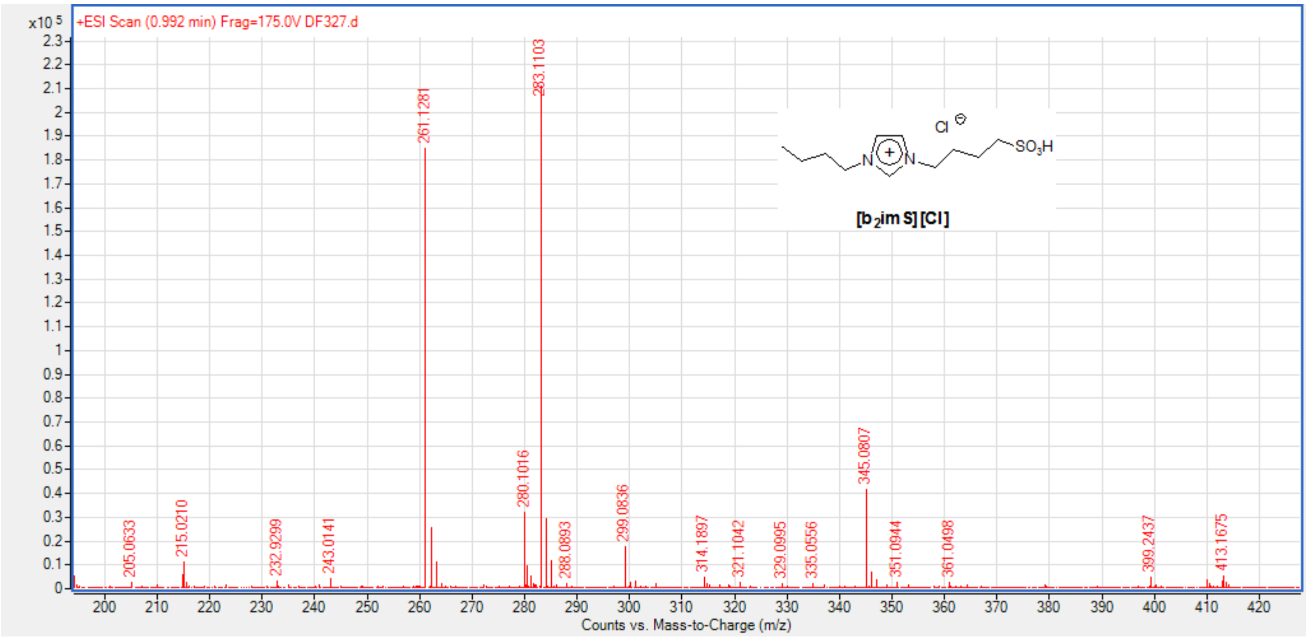
+ESI Scan (0.992 min) Frag=175.0V DF327.d
x10 5
Counts vs. Mass-to-Charge (m/z)
261.1281
283.1103
280.1016
345.0807
299.0836
215.0210
205.0633
232.9299
243.0141
288.0893
314.1897
321.1042
329.0995
335.0556
351.0944
361.0498
399.2437
413.1675
[b2im S][Cl]

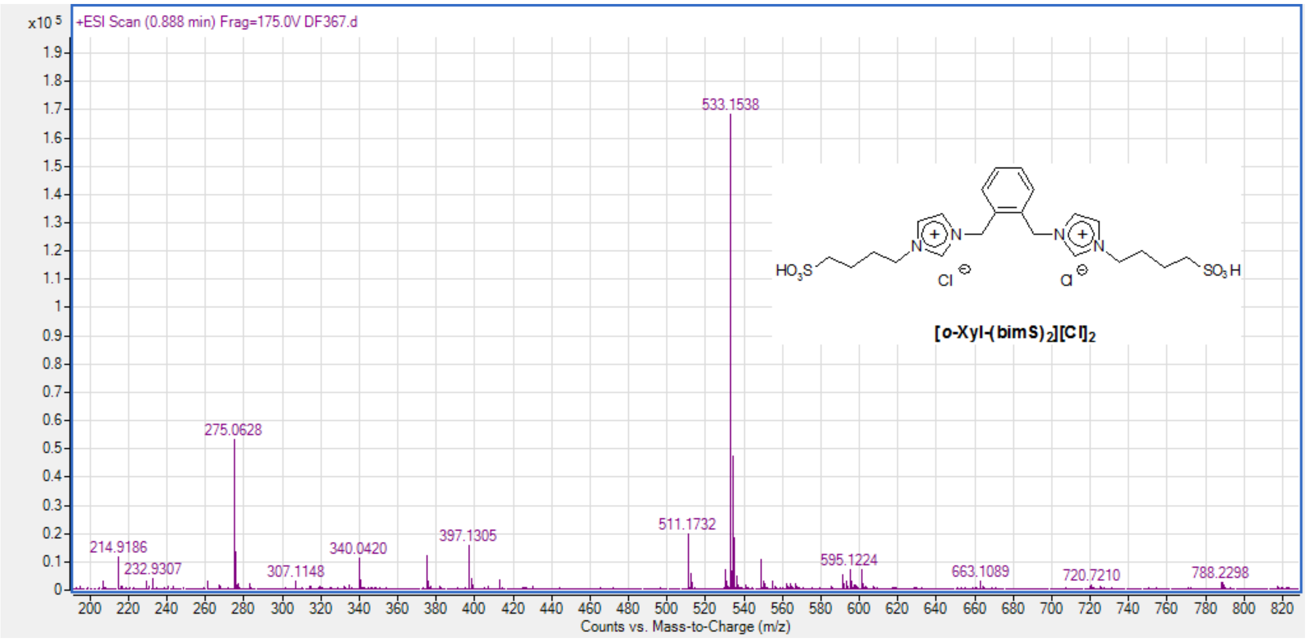
+ESI Scan (0.888 min) Frag=175.0V DF367.d
x10 5
Counts vs. Mass-to-Charge (m/z)
533.1538
275.0628
511.1732
397.1305
340.0420
214.9186
232.9307
307.1148
595.1224
663.1089
720.7210
788.2298
[o-Xyl-(bimS)2][Cl]2

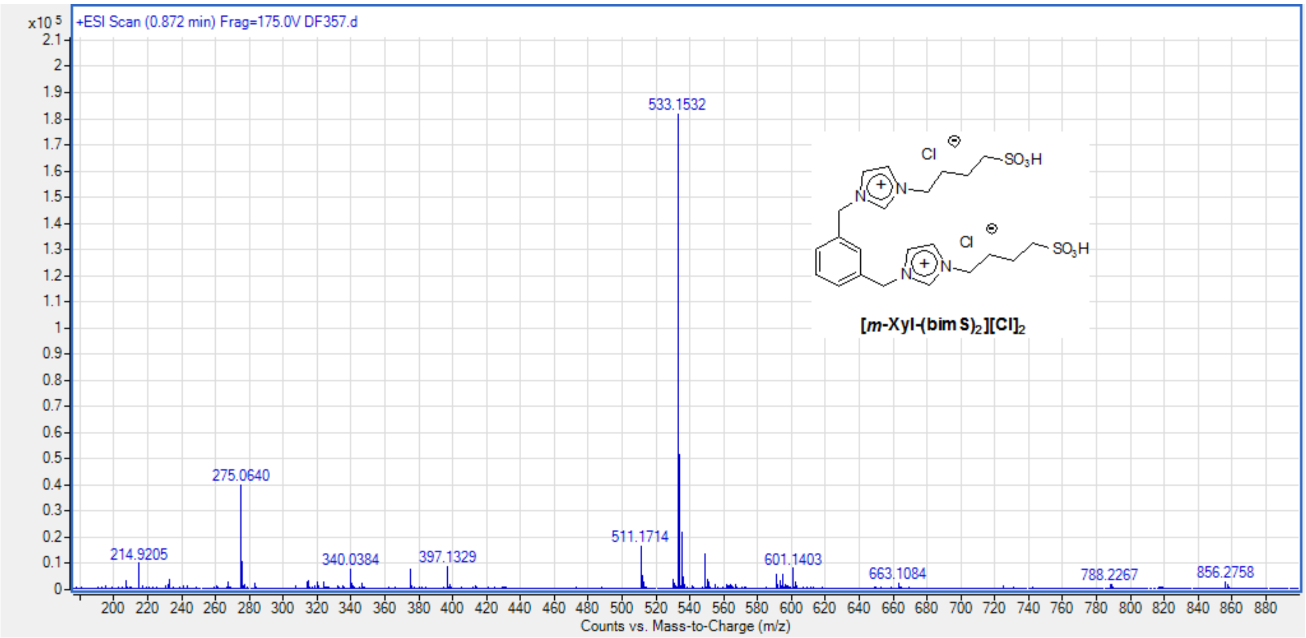
x10 5
+ESI Scan (0.872 min) Frag=175.0V DF357.d
533.1532
275.0640
214.9205
340.0384
397.1329
511.1714
601.1403
663.1084
788.2267
856.2758
Counts vs. Mass-to-Charge (m/z)
[m-Xyl-(bimS)2][Cl]2

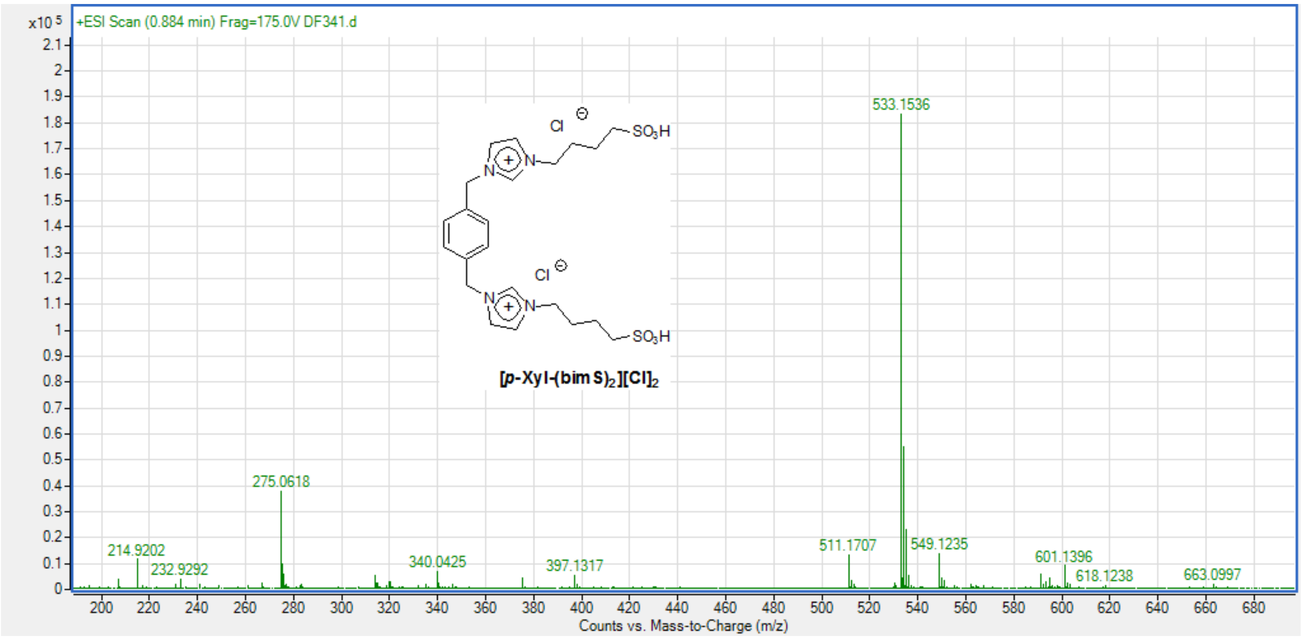
x10 5
+ESI Scan (0.884 min) Frag=175.0V DF341.d
533.1536
275.0618
214.9202
232.9292
340.0425
397.1317
511.1707
549.1235
601.1396
618.1238
663.0997
Counts vs. Mass-to-Charge (m/z)
[p-Xyl-(bimS)2][Cl]2

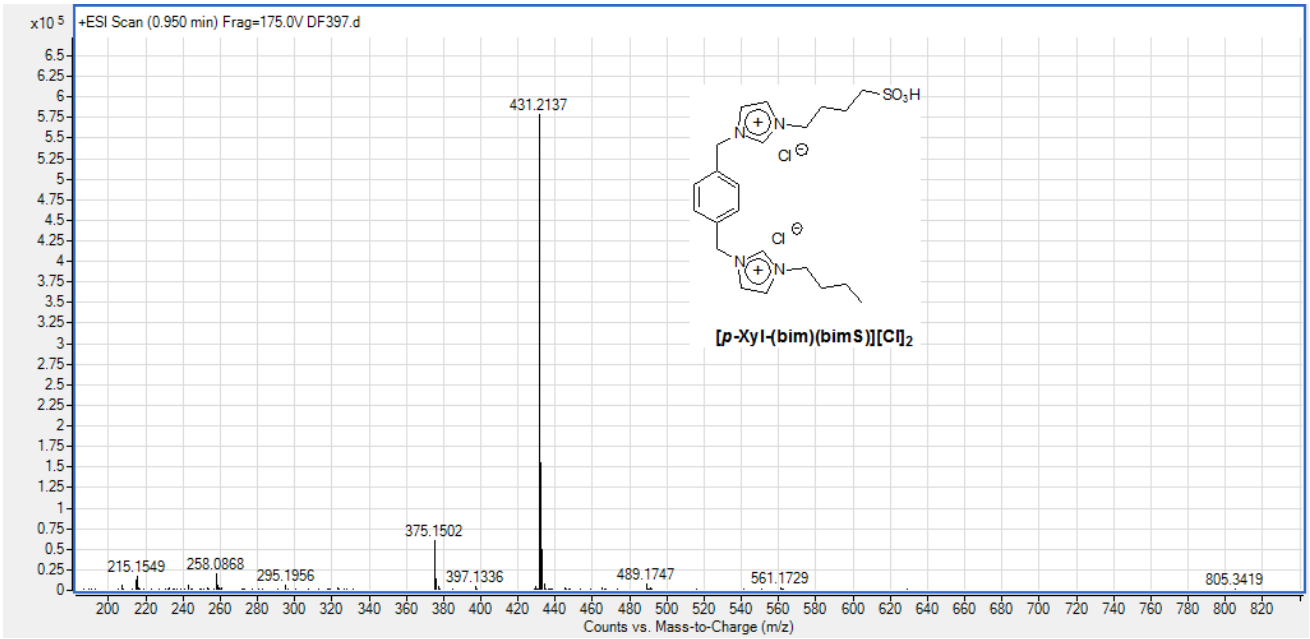

Figure S4. ESI Mass spectra of the TSILs

Table S1. Yields in 5-HMF from fructose and sucrose, at 60°C, with and without performing hydrolysis as a function of **[b$_2$imS][Cl]** amount.

Substrate	[b$_2$imS][Cl] (mol %)	Yield without hydrolysis[a] (%)	Yield after hydrolysis[a] (%)
Fructose[b]	5	9	8
	10	22	26
	15	25	35
	17	28	39
	20	27	44
	25	24	51
	30	18	62
	40	12	61
	50	8	59
Sucrose[c]	5	-	-
	10	22	25
	15	22	25
	20	16	33
	25	9	33
	30	7	35
	40	4	33
	50	-	32

[a] Yields are reproducible within ±2 %. [b] t = 105 min. [c] t= 150 min.

Table S2. Yields in 5-HMF from fructose, at 60°C and after 105 minutes, as a function of catalyst amount.

Catalyst	Catalyst amount (mol %)	Yield (%)[a]
**[*o*-Xyl-(bimS)$_2$][Cl]$_2$**	5	-
	7	18
	10	40
	15	55
	20	63
	25	67
	30	66
	35	65
	40	65
**[*m*-Xyl-(bimS)$_2$][Cl]$_2$**	15	15
	20	27
	25	35
	30	40
	35	43
	40	45
	45	49.
	50	50
	55	51
**[*p*-Xyl-(bimS)$_2$][Cl]$_2$**	5	-
	10	-
	15	10
	20	32
	25	45
	30	52
	35	57
	40	63
	45	57

[a] Yields are reproducible within ±2 %.

Table S3. Yields in 5-HMF from sucrose, at 60 °C and after 150 minutes, as a function of catalyst amount.

Catalyst	Catalyst amount (mol %)	Yield (%)[a]
[*o*-Xyl-(bimS)$_2$][Cl]$_2$	5	-
	10	5
	15	25
	20	30
	25	32
	30	34
	35	32
	40	31
[*m*-Xyl-(bimS)$_2$][Cl]$_2$	10	-
	15	11
	20	23
	25	31
	30	31
	35	30
	40	31
	45	32
	50	32
	55	31
[*p*-Xyl-(bimS)$_2$][Cl]$_2$	10	-
	15	10
	19	22
	20	29
	25	29
	30	30
	35	29

[a] Yields are reproducible within ±2 %.

Table S4. Yields in 5-HMF from fructose, at 60°C in the presence of 20 mol % of catalyst, as a function of time.

Catalyst	Time (min)	Yield (%)[a]
**[$b_2$imS][Cl]**	15	18
	30	24
	45	32
	60	39
	75	43
	90	47
	105	62
	120	59
**[*o*-Xyl-($bimS)_2$][$Cl]_2$**	15	19
	30	31
	45	39
	60	43
	75	45
	90	49
	105	53
	120	55
	135	58
	150	60
	165	59
	180	57
**[*m*-Xyl-($bimS)_2$][$Cl]_2$**	15	21
	30	36
	45	45
	60	52
	75	54
	90	59
	105	49

[a] Yields are reproducible within ±2 %.

Table S5. Yields in 5-HMF from sucrose, at 60°C in the presence of 20 mol % of catalyst, as a function of time.

Catalyst	Time (min)	Yield (%)[a]
**[$b_2$imS][Cl]**	15	15
	30	20
	45	26
	60	26
	75	29
	90	32
	105	31
	120	29
**[*o*-Xyl-$(bimS)_2$][$Cl]_2$**	15	-
	30	5
	45	10
	60	14
	75	18
	90	20
	105	24
	120	27
	135	30
	150	31
	165	28
	180	
**[*m*-Xyl-$(bimS)_2$][$Cl]_2$**	30	10
	45	16
	60	18
	75	23
	90	25
	105	29
	120	26

[a] Yields are reproducible within ±2 %.

.
